# Anatomically constrained volumetric smoothing enhances fMRI reliability while avoiding smoothing artifacts

**DOI:** 10.3389/fnimg.2026.1753534

**Published:** 2026-05-01

**Authors:** David G. Ellis, Michele R. Aizenberg

**Affiliations:** Department of Neurosurgery, University of Nebraska Medical Center, Omaha, NE, United States

**Keywords:** fMRI, spatial smoothing, anatomically constrained smoothing, task activation, functional connectivity, volumetric smoothing artifacts

## Abstract

**Introduction:**

Smoothing fMRI data prior to analysis is a fundamental and widely used technique to increase sensitivity. Unconstrained smoothing can also reduce the spatial specificity of the analysis by introducing artifacts in the data. This study tested the effects of smoothing on the reliability and accuracy of both task fMRI and resting state data. The effects of unconstrained smoothing were compared to those of an anatomically constrained smoothing method, which prevents smoothing across the white and gray matter surfaces of the cortex.

**Methods:**

Unconstrained Gaussian smoothing and anatomically constrained smoothing were applied to simulated data, a sensory task fMRI dataset, a precision fMRI motor task mapping dataset, and a resting state fMRI dataset. Smoothing-related artifacts were tested for and compared between the smoothing methods, and the effects of the smoothing methods on the reliability and accuracy were measured.

**Results:**

In the experiments with simulated data, unconstrained Gaussian smoothing demonstrated decreased accuracy and increased white matter activation compared to constrained smoothing. In the sensory task activation analysis, both Gaussian and constrained smoothing increased the reliability of the sensory task fMRI activations, but Gaussian smoothing increased the percentage of active voxels in the white matter relative to constrained smoothing (*p* < 0.001). Relative to constrained smoothing, Gaussian smoothing with FWHM > 3 mm also decreased the accuracy of motor mapping results from individual sessions to the precision maps (*p* < 0.001). With cluster significance thresholding, mean false positive voxel percentages remained below 5% for both methods across the tested kernel widths. Both Gaussian and constrained smoothing demonstrated a biasing effect on the resting state connectivity of nearby regions and on the graph theory metrics of the functional connectomes.

**Conclusion:**

This study showed that unconstrained Gaussian smoothing spreads activation across cortical boundaries, increases white matter activation, and biases graph theory connectivity metrics. Anatomically constrained smoothing reduced some of these smoothing artifacts while still increasing reliability and may be a reasonable alternative to unconstrained Gaussian smoothing.

## 1 Introduction

Smoothing fMRI data prior to analysis is a fundamental and widely used technique to enhance the signal-to-noise ratio of the data and improve the sensitivity and statistical power of the analysis (Mikl et al., 2008; Worsley and Friston, 1995). Smoothing aggregates signal across multiple neighboring voxels or data points to boost signal and dampen noise. While effective at increasing the sensitivity of fMRI analysis, smoothing can reduce spatial specificity (Mikl et al., 2008). The increased sensitivity allows for better detection of activation while the reduction in spatial specificity makes the precise region or location of the activation more ambiguous. In some anatomically homogeneous locations, such as voxels contained within the same gyrus, the precise spatial location of activation within that gyrus may be of less concern as long as the correct gyrus of activation can be discerned. However, volumetric smoothing can also aggregate signal across anatomically distinct structures due to their close spatial proximity in Euclidean space (Brodoehl et al., 2020; Jo et al., 2007). This includes smoothing across the complex sulcal folds of the cortex and blurring of signal across the white-gray matter interface (Yang et al., 2025; Coalson et al., 2018). Such smoothing artifacts may distort estimates of task activation clusters as well as functional connectivity measures (Mikl et al., 2008; Alahmadi, 2021; Candemir, 2023; Triana et al., 2020).

Surface-based fMRI analysis techniques represent the current state-of-the-art for circumventing volumetric smoothing artifacts. By constraining the modeling and analysis of activity to the unfolded cortical surface, this approach inherently respects the geodesic distance along the cortex, effectively preventing signal from bleeding across sulci (Brodoehl et al., 2020; Hagler et al., 2006; Andrade et al., 2001). Despite their proven benefits and increasing adoption, many established neuroimaging software tools still lack integration of surface-based workflows. Surface-based fMRI analysis requires complex preprocessing pipelines for cortical reconstruction and projection, as well as specialized file formats (e.g., CIFTI) that are less intuitive to inspect, access, and manipulate than the voxel grid used in 3D images. Performing surface-based fMRI analysis often requires researchers to rely on a small set of specialized tools, which may or may not support their preferred workflows or provide desired options for customization. These practical barriers represent significant hurdles to the broader adoption of surface-based analyses in fMRI.

**Figure 1:**
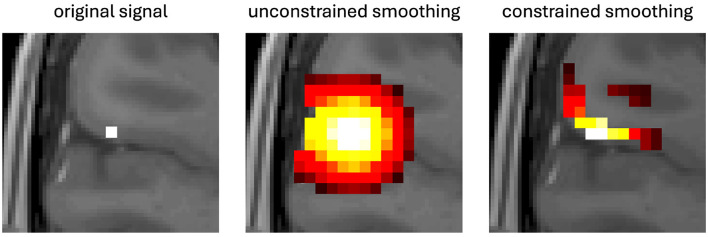
Illustration of constrained vs. unconstrained smoothing with the original signal shown as a white dot on the left, unconstrained smoothing of the original signal shown in the middle, and constrained smoothing shown on the right. The unconstrained volumetric approach smooths signal to neighboring voxels causing signal from one gyrus to spread across the sulcus into a separate gyrus as well as into the white matter of the original gyrus. By contrast, constrained smoothing uses the surfaces of the cortex to constrain the smoothing of the original signal and prevent the signal from smoothing into the neighboring gyrus or white matter.

A potential alternative approach to surface-based smoothing could be to constrain the volumetric smoothing of fMRI data based on the cortical anatomy. Recent research by Behjat et al. demonstrated a novel methodology for preventing the volumetric smoothing of the cortex from smoothing across proximal but functionally distinct cortical gyri as well as the white-gray matter interface (Behjat et al., 2021). Their approach modeled cortical voxels as nodes of a graph with connections being the neighboring voxels. After building the graph the edges were then selectively pruned such that any connection that crossed the white matter or pial surfaces was removed. Volumetric spatial smoothing was then performed using graph signal processing techniques on the pruned graph. Performing smoothing on the pruned graph inhibited data from being smoothed across the anatomical boundaries and prevented common volumetric smoothing artifacts. Figure 1 illustrates how this constrained smoothing approach avoids smoothing artifacts caused by typical unconstrained volumetric smoothing.

Building on the work by Behjat et al., we created a novel implementation of volumetric constrained smoothing (Behjat et al., 2021). Our implementation allowed us to directly compare the qualitative and quantitative effects of Gaussian and constrained smoothing at similar smoothing kernel widths on simulated data as well as real-world task and resting state fMRI data. This study demonstrates the negative effects of unconstrained volumetric smoothing while showing that constrained volumetric smoothing is a promising alternative for avoiding these smoothing artifacts.

## 2 Methods

### 2.1 Constrained smoothing

We developed a tool to perform anatomically constrained volumetric smoothing that builds a graph of the nodes and connections of the brain voxels, prunes the graph to remove connections passing through a cortical surface (either pial or white), and then applies smoothing to the fMRI data based on the graph. As compared to (Behjat et al. 2021), our implementation:

1. Uses ray tracing to quickly evaluate surface intersections rather than iterating through each connection.
2. Allows the user specify the desired smoothing kernel width in terms of full-width at half-maximum (FWHM) of a Gaussian kernel. The heat kernel smoothing parameter is then iteratively optimized to approximate the desired FWHM, allowing for more intuitive implementation. This implementation also allows for the direct comparison between constrained and unconstrained smoothing at similar smoothing kernel widths.
3. Is written in Python which, unlike MATLAB, does not require a license or subscription fee. We provide a Docker image to allow for easy implementation by other researchers (see the Acknowledgments for more details).

#### 2.1.1 Graph construction and pruning

The first step in anatomically constrained smoothing is to build a graph network where each node in the graph represents a voxel and each edge in the graph represents a connection to a neighboring voxel. This graph was built based on a binary mask of the brain dilated by 3 voxels to ensure no potential brain voxels were discarded due to poor brain mask segmentation. Each voxel of the dilated brain mask was then considered a node in the graph. Edges were added to the graph such that each node was connected to all 26 of its neighbors in 3D voxel space, as in (Behjat et al. 2021). To constrain the graph from allowing signal to be smoothed across cortical boundaries, edges were pruned that crossed either the pial or white surface as reconstructed by FreeSurfer (Fischl, 2012). Rather than iterating through each connection and checking for intersections, we used ray tracing (Dawson-Haggerty, 2019; Wald et al., 2014) to quickly and simultaneously check for the intersections between all connections in the graph and a given surface. This allowed for graph pruning to be completed in about 2 min.

**Figure 2:**
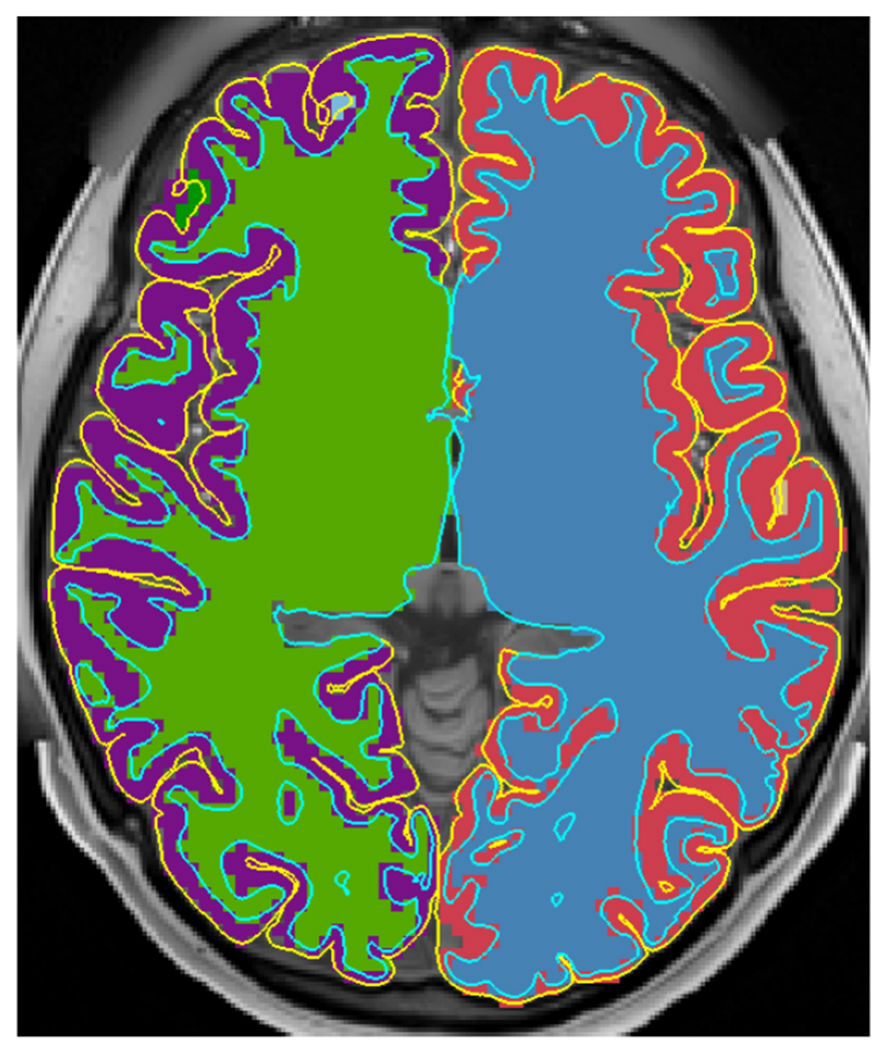
Connected components of the graph after edge pruning, excluding the CSF and background. The white matter surfaces are shown in blue and the pial surfaces are shown in yellow. Edge pruning effectively separates both the left and right hemispheres and the white and gray matter components.

After pruning the connections the graph was broken up into its constituent separately connected components. The edge pruning process effectively separated the white and gray matter and for each hemisphere, splitting the brain into four components of interest: left/right white matter and left/right gray matter. As shown in Figure 2, the white matter and gray matter components represent the four largest components of the graph after excluding the background/CSF component. As these components are not connected to each other, they can be smoothed independently. We implemented a heuristic check to make sure the expected number of connected components was present following pruning.

#### 2.1.2 Equivalent parameter estimation and smoothing

Tools for fMRI processing often allow the user to specify smoothness in terms of the full-width at half maximum (FWHM) in millimeters of the Gaussian kernel. Using FWHM allows for an intuitive understanding of the size of the smoothing kernel being applied to the data. Unfortunately, for the large graphs (> 40 million nodes) used in this project computing the Gaussian kernels for each node proved computationally infeasible for large kernels. Instead, we utilized the heat smoothing kernel as was done previously (Behjat et al., 2021). Heat kernel smoothing allows for quick smoothing of graph signal data, but, instead of FWHM, uses a less intuitive parameter τ to control the extent of smoothing within the graph. To allow the user to specify an approximate smoothing kernel width in terms of FWHM for the smoothing to be applied, we implemented an optimization method to estimate a τ value for heat kernel smoothing that best approximated a Gaussian kernel of the desired width. Inspired by (Hagler et al. 2006), normally distributed random values for each node of the graph in a given component were generated. Heat kernel smoothing was then applied to the random data using an initial estimate for τ. After smoothing, the following equation, adapted from (Hagler et al. 2006), was used to estimate the smoothing width of the smoothed random data in the component:


$$\begin{array}{l} FWHM_{\text{graph}}=dn\sqrt{\frac{-2\ln\;2}{\ln\left(1-\mathrm{var}\left(ds\right)\mathrm{var}\left(s\right)\right)}}, \end{array}$$ (1)

where *dn* is the average distance between neighbors in the graph, *var*(*ds*) is the variance in the difference in signal between neighbors within the graph, and *var*(*s*) is the variance of the signal over all nodes of the graph.

Based on the estimated FWHM, τ was updated using gradient descent and the unsmoothed random data were re-smoothed with the updated τ. This process was iteratively repeated until a value of τ was found that smoothed the random data to the approximate desired FWHM. This optimization was repeated for each connected component of the graph. The final output from this optimization procedure was a value of τ for each component that would achieve the user-specified smoothing width in terms of estimated FWHM. This optimized value for τ was then used to smooth the fMRI data for its given component.

Each component was smoothed separately using the graph signal processing (Shuman et al., 2013) method of heat kernel smoothing with PyGSP (Defferrard et al., 2017).

### 2.2 Experiments

#### 2.2.1 Simulation study

We hypothesized that constrained smoothing would perform better than Gaussian smoothing at isolating cortical regions of interest in the presence of white noise. To test this hypothesis, we utilized the FreeSurfer cortical parcellation label maps to isolate 68 unique cortical regions from a single subject in the Midnight Scan Club (MSC) dataset (Gordon et al., 2017). The cortical parcellation maps were derived from a single T1w image from that subject [TR = 2.4 s, TE = 3.74 ms, and isotropic resolution equal to 0.8 mm (Gordon et al., 2017)]. For each cortical region, a 3D mask of that region was created and served as the ground truth mask. For each of these regions, a noisy image was generated where the region of interest was set to an amplitude of 4 and random white noise was added with a mean of zero and a standard deviation of 2. The noisy images were then smoothed using both Gaussian and constrained smoothing. The accuracy of the smoothed images was compared to the ground truth mask using both Dice score and Pearson correlation. For Dice scores and active voxel counts, a percentile threshold was used to isolate a fixed number of active voxels that was equal to the number of voxels in the ground truth mask.

#### 2.2.2 Sensory task activation

In this experiment, we sought to understand the effects of Gaussian and constrained smoothing on sensory task fMRI mapping. We hypothesized that the primary location of activation for a left-hand sensory task should be in the gray matter of the right postcentral gyrus, and that Gaussian smoothing would reduce the spatial specificity of the activation resulting in increased activation in the precentral gyrus and the white matter. Relative to Gaussian smoothing, anatomically constrained smoothing, if effective, should show a decreased effect on the size of activation in these areas.

To test if either Gaussian or constrained smoothing would spread sensory activation beyond the sensory cortex, we used data from a public dataset of 10 subjects who completed fMRI sensory stimulation mapping (Reddy et al., 2024). Each of the 10 subjects completed two runs of a block design sensory stimulation task, where a brushing stimulation was applied to the left-hand. The data was acquired with a Siemens 3T Prisma MRI, voxel size of 1.7 mm × 1.7 mm × 4 mm, TR of 2.2 s, and 235 volumes per run for BOLD scans, and TR = 2.17 s, TE = 1.69/3.55/5.41 ms with 1 mm isotropic voxel size for T1-weighted multiecho MPRAGE scans. The data was preprocessed with fmriprep (version 24.1.1) (Esteban et al., 2019). Each run was smoothed using either volumetric Gaussian smoothing or our proposed constrained smoothing approach with FWHM ranging from 3 mm to 12 mm in 1 mm increments. Data was also processed with no smoothing as a baseline. Following smoothing, activations were computed using FSL FEAT (version 6.0.7.1) with multiplicative mean intensity normalization, high pass temporal filtering, and local autocorrelation correction (Jenkinson et al., 2012; Woolrich et al., 2001).

Any voxels with z-statistic values from FSL FEAT above 3.1 were considered activated voxels, as is the default in FSL, except without cluster-wise significance correction. A static threshold was utilized to compare activation extent, localization, and reliability while avoiding introducing an additional source of variation from a particular multiple-comparison correction procedure.

To understand the effect of each smoothing method on the size of activations in distinct regions of interest, the number of activated voxels in the precentral gyrus, postcentral gyrus, gray matter, and white matter were counted. These regions of interest were defined from segmentations produced by FreeSurfer (Fischl, 2012) and fmriprep (Esteban et al., 2019).

A generalized linear mixed model (GLMM) was used to compare the effects of Gaussian and constrained smoothing on the number of activated voxels in the regions of interest with the subject and run considered random effects. The number of active voxels was modeled as counts using a negative binomial distribution with a log link function. The smoothing kernel width in terms of FWHM and the interaction between the method and smoothing kernel width were the independent variables in the model.


$$\begin{array}{l} \begin{array}{cc} active_{\text{ROI}} & =\beta_{0}+\beta_{1}\text{FWHM}+\beta_{2}\text{method}+\beta_{3}\times \text{method}\\ \;\times \text{FWHM}+u_{\text{subj}\left[k\right]}+u_{\text{run}\left[l\right]}+\epsilon \end{array} \end{array}$$ (2)

Where active_ROI_ is the number of active voxels for the ROI of interest, β_0_ is the global intercept, β_1_ is the estimated slope of the effect of the smoothing kernel width being applied, β_2_ is the adjusted baseline intercept for constrained smoothing as compared to Gaussian smoothing (with method = 0 for Gaussian and 1 for constrained), β_3_ is the interaction between the smoothing kernel width and smoothing method being used, *u*_subj[*k*]_ is the random effect for subject *k*, *u*_run[*l*]_ is the random effect for run *l*, and ϵ is the residual error. Separate models were fit for each region. To describe the effect of increasing smoothing on the number of active voxels, we reported the incidence rate ratios per 1 mm increase in FWHM. The incidence rate ratio was exp(β_1_) for Gaussian smoothing and exp(β_1_+β_3_) for constrained smoothing.

We also analyzed the effect of smoothing on the reliability of the activations by using Dice similarity coefficient (DSC) to measure the overlap of active voxels between runs. The average reliability as measured by DSC was analyzed for all subjects with no smoothing as well as with smoothing kernel widths from FWHM of 3 mm to FWHM of 12 mm in 1 mm increments. We then fit the GLMM from Equation 2 again, this time modeling DSC as the response variable with a normal distribution and identity link function (instead of a negative binomial with a log link function as described previously) and without including the run as a random effect.

#### 2.2.3 Motor task activation accuracy

To test the accuracy of fMRI activations under different smoothing approaches and kernel widths, we utilized data from the Midnight Scan Club dataset, which includes 10 highly sampled subjects (Gordon et al., 2017). Each of the subjects underwent 20 runs of a motor task fMRI scanning, which involved left/right finger tapping, left/right toe tapping, and tongue movement in a block design (Gordon et al., 2017). The task fMRI images were acquired using a TR of 2.2 s, a TE of 27 ms, and an isotropic resolution of 4 mm (Gordon et al., 2017). Additionally, T1-weighted (T1w) images were acquired for each subject with a TR of 2.4 seconds, a TE of 3.74 ms, and an isotropic resolution of 0.8 mm (Gordon et al., 2017). Data was preprocessed using fmriprep (version 23.2.0) (Esteban et al., 2019).

We utilized FSL FEAT (Jenkinson et al., 2012) with no smoothing to create activation maps for each motor task movement and run. To create a target task map for each subject, we averaged the maps across all runs and sessions. This average map served as a pseudo-ground truth activation map for measuring the accuracy of maps from a smaller number of runs. This pseudo-ground truth is an aggregation of noisy individual run data, and therefore is not a biological or statistical gold standard. However, averaging over multiple runs should provide a better representation of the true relationship between the task and the BOLD response than any of the individual runs. Additional run-level activation maps were also computed using FSL after smoothing with either Gaussian or anatomically constrained smoothing. We then measured the accuracy using DSC, Pearson r correlation, and mean absolute error (MAE) of each smoothing method and kernel width (FWHM = 3, 6, 9, or 12 mm). For the comparison using DSC, the binary positive predictions were derived from the z-statistic maps by applying a threshold of z-statistic > 3.1 without cluster significance correction. The accuracy was measured for each set of 2 runs taken in the same session as compared to the pseudo-ground truth map, which was the average across all runs with no smoothing applied. Paired sample t-tests were used to compare the average errors between methods.

#### 2.2.4 False positive voxels

Performing surface-constrained, and thus anisotropic, smoothing produces smoothed data that likely violates the Gaussian random field assumptions of the parametric statistical models used by task fMRI software packages (such as FSL FEAT) (Eklund et al., 2016). Previous research has shown that the violation of the Gaussian random field assumptions can lead to high false positive rates in fMRI studies (Eklund et al., 2016). Therefore, we sought to analyze and compare the percentage of false positive voxels with both constrained and unconstrained Gaussian smoothing. To study the false positive voxel percentage, we performed a task fMRI analysis on resting state (i.e., task-less) data (similar to Eklund et al., 2016). Because the data was not acquired with any task being performed, any voxels detected as being positively correlated with the task design can be considered false positive voxels. Resting state data from the Human Connectome Project in Aging (HCPA) dataset was used (Bookheimer et al., 2019). The subjects in the HCPA dataset were healthy adults 30-100 years of age. Image acquisition included 4 resting state scans per subject, with each scan lasting 6.5 minutes and containing 488 volumes for a total of 26 minutes and 1952 volumes per subject. Resting state data was acquired with an isotropic voxel size of 2 mm and a TR of 0.8 seconds. T1w image data was acquired with an isotropic resolution of 0.8 mm (TR = 2.5 s, TE = 2.22 ms).

Data was preprocessed using fmriprep (version 24.1.1) (Esteban et al., 2019). To correct for potential artifacts in the data, bandpass filtering (0.01 to 0.2 Hz) and confound regression were performed such that 24 motion confounds along with 12 white matter, CSF, and global signal confounds were regressed from the time series. Additionally, motion scrubbing was performed such that volumes with a framewise displacement (FD) greater than 0.5 mm were removed from the data (Power et al., 2014). Data was smoothed with constrained and Gaussian smoothing, at FWHM values of 3, 6, 9, and 12 mm, and then processed with FSL FEAT. In this analysis, we used a block task design and analysis in FSL FEAT, with each block lasting 30 s. Positive (i.e., active) voxels were considered using the default FSL threshold of z-statistic > 3.1, both with and without cluster significance thresholding applied (*p* = 0.05) (Jenkinson et al., 2012). The false positive voxel percentage was defined as the number of positive voxels out of the total number of voxels in the MNI brain mask. The mean false positive voxel percentage per smoothing kernel width was compared between Gaussian and constrained smoothing using paired t-tests. In order to identify potential anatomical regions with higher false positives, the average false positive percentage in MNI space was plotted for qualitative comparison between smoothing kernel widths and methods.

#### 2.2.5 Connectivity metrics

Beyond task activation fMRI, volumetric smoothing has been shown to alter connectivity measures in resting state fMRI (rsfMRI) studies (Triana et al., 2020; Candemir, 2023; Alahmadi, 2021). To better understand the differential effects of constrained and unconstrained smoothing on ROI to ROI connection strength and graph network measures, we studied resting state data from subjects in the HCPA dataset, as described previously. Subjects with greater than 20% of their resting state frames removed due to motion scrubbing were excluded from the analysis due to excessive motion. Following preprocessing, data was processed with no smoothing, a Gaussian smoothing kernel ranging from 3 mm to 12 mm FWHM in 3 mm increments, or constrained smoothing with the same estimated smoothing kernel widths as that of the Gaussian approach.

In general, regions of the brain that are spatially close tend to be more functionally associated and have greater functional connectivity (Oligschläger et al., 2017). We hypothesized that unconstrained smoothing artificially inflates functional connection strength between nearby regions beyond that which is inherent due to the relationship between structure and function in the brain. To test this hypothesis, we used the Schaefer/Yeo 1,000 parcels 7 networks atlas (Schaefer et al., 2017), and derived Euclidean distances from the centroid of each parcel to the centroid of every other parcel in the atlas. For each subject, all four resting state runs were concatenated and the correlation of the BOLD data between each region of interest from the atlas was computed to create the connectivity matrix. A separate connectivity matrix was computed for each smoothing condition and the correlation values were Fisher z-transformed prior to analysis.

To study the effect of smoothing on resting state connectivity strength analyses we used a generalized additive mixed model (GAMM). Using a GAMM allows for the interaction between distance and smoothing to be non-linear in relation to connectivity. We expected that smoothing would have a non-linear relationship to connectivity as smoothing likely increases the connectivity of regions that are spatially proximal, but that this effect likely goes to zero for spatially distant regions. We defined our GAMM model as


$$\begin{array}{l} zcorr_{k,ij}^{\left(M,H\right)}=\beta_{0}+f_{M,H}\left(d_{ij}\right)+u_{\text{subj}\left[k\right]}+\epsilon_{k,ij}^{\left(M,H\right)}, \end{array}$$ (3)

where β_0_ is the global intercept, $zcorr_{k,ij}^{\left(M,H\right)}$ is the Fisher z-transformed correlation value between regions *i* and *j* for subject *k* under the condition where smoothing is applied with method *M*∈{*Gaussian, Constrained*} and FWHM smoothing level *H*∈{0, 3, 6, 9, 12} (in mm), *d*_*ij*_ is the Euclidean distance between the centroids of regions *i* and *j* in template space, and *u*_subj[*k*]_ represents the random effect for subject *k*. Separate splines, represented by *f*_*M, H*_, were fitted to describe the relationship between the ROI to ROI distance and connectivity for each interaction between each smoothing method and FWHM smoothing level. Random effects for each ROI to ROI pair were not included because each ROI pair has a fixed distance *d*_*ij*_ creating a situation where the ROI pair random effect would perfectly confound the effect on distance. Therefore, including ROI pair random effects would prevent estimation of the effect of distance on the connectivity, which was the primary focus of this analysis.

Additionally, we studied the effects of smoothing on graph network connectivity measures. Using the Schaefer/Yeo 1,000 Parcels 7 Networks atlas (Schaefer et al., 2017), we created graph connectivity networks based on Pearson r correlation values. The graph density was set to 20% such that only the top 20% of correlations were considered connections in the graph. Clustering coefficient, global efficiency, local efficiency, and modularity were then computed for each smoothing method at FWHM values of 3, 6, 9, and 12 mm. We then used paired t-tests to compare Gaussian and constrained smoothing to each other as well as against no smoothing.

## 3 Results

### 3.1 Simulation study

**Figure 3:**
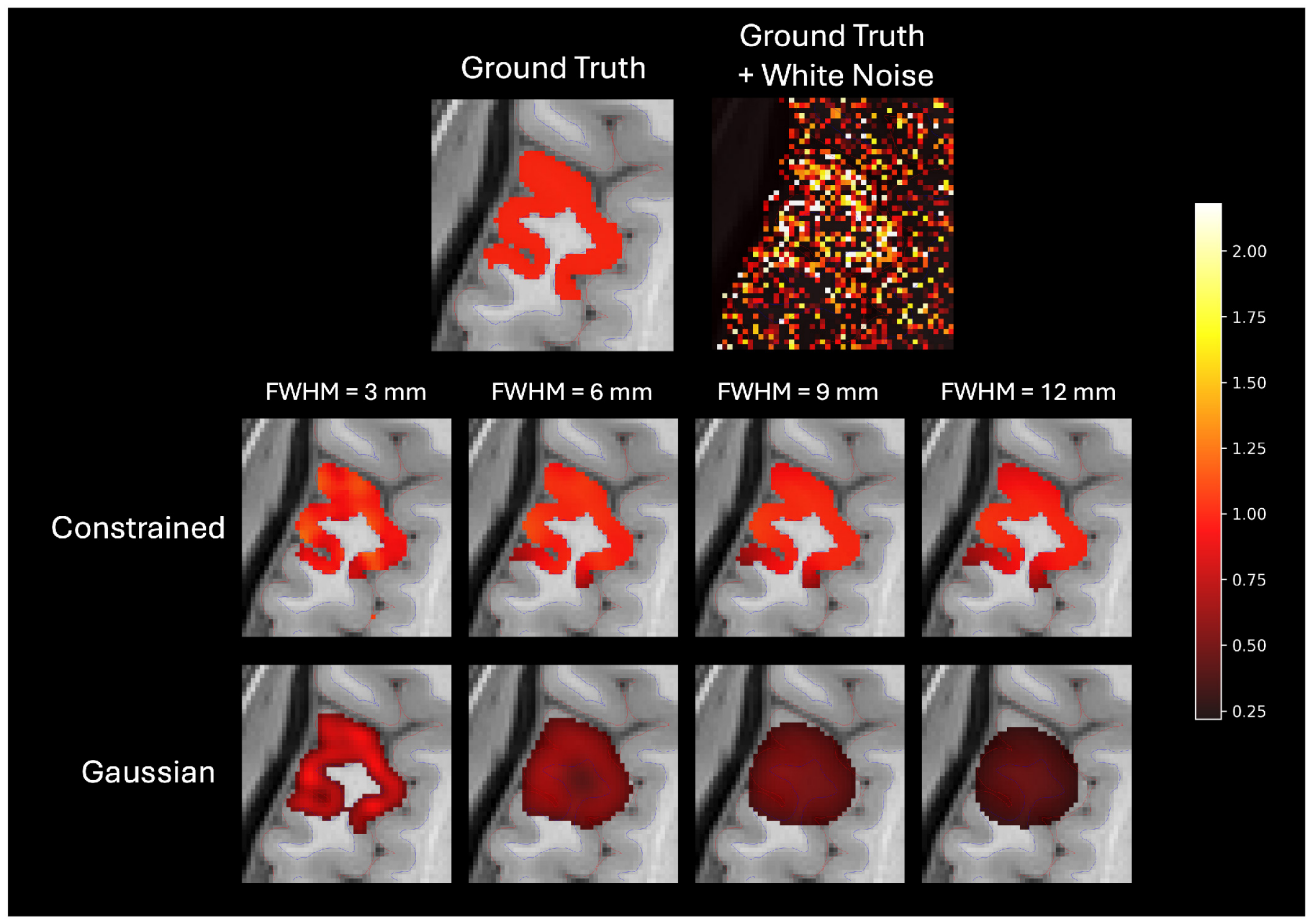
Example of a single simulation with arbitrary units where random white noise was added to a ground truth cortical region producing a noisy image. This image was then smoothed with either Gaussian or constrained smoothing with FWHM ranging from 3 mm to 12 mm. As shown in the example, constrained smoothing retains the structure of the cortical region while Gaussian smoothing does not.

To test our hypothesis that constrained smoothing would perform better than Gaussian smoothing at isolating cortical regions of interest, we generated 68 cortical region masks and added white noise to them. The noisy images were smoothed using both Gaussian and constrained smoothing and the accuracy of the smoothed images was compared to the ground truth masks. As shown in Figure 3, constrained smoothing accurately recovered and retained the shape of the original regions of activation, while Gaussian smoothing tended to create smoothing artifacts by smoothing across boundaries.

**Figure 4:**
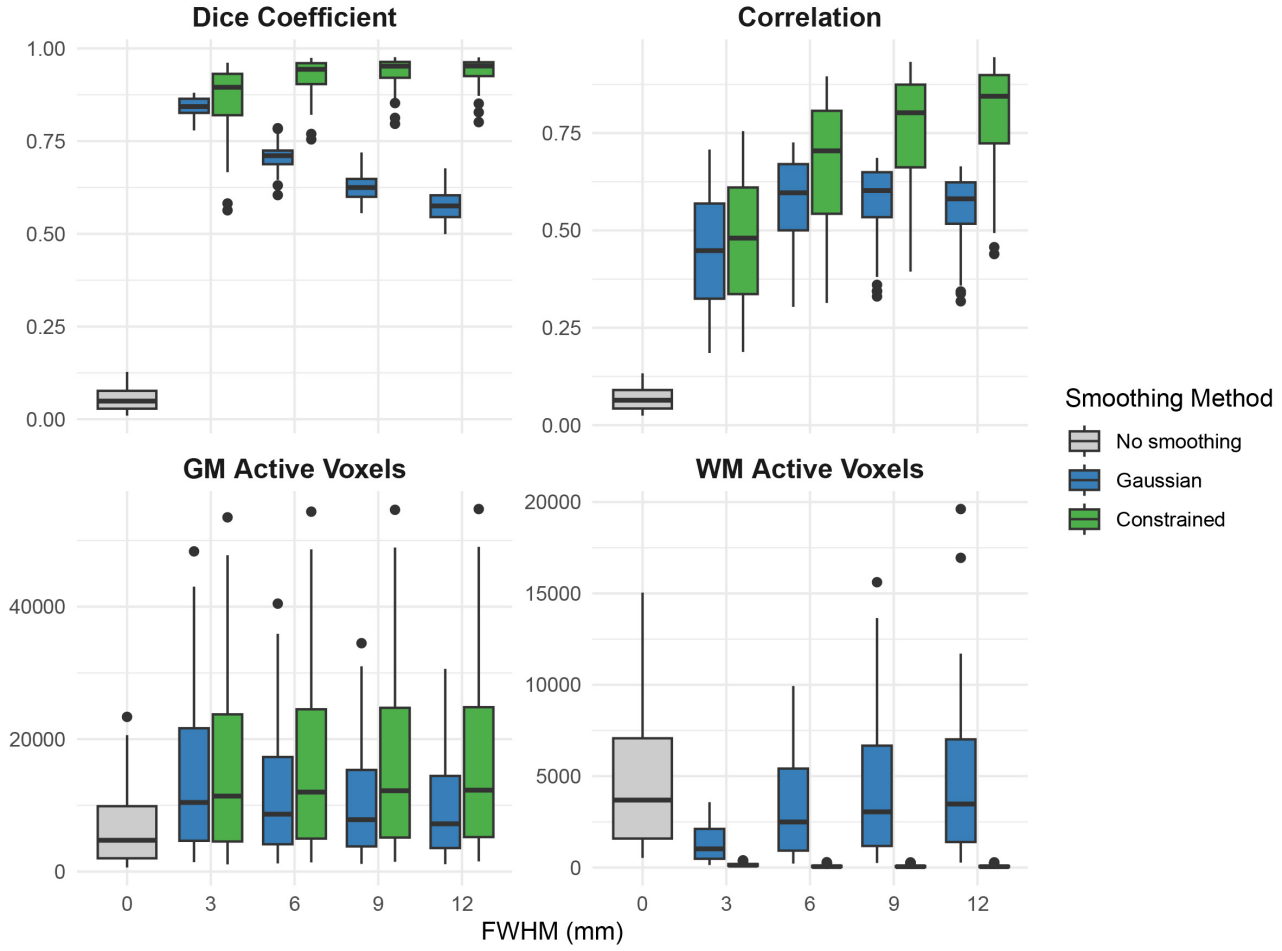
**(Top left)** Dice similarity score coefficients, **(top right)** Pearson correlations, **(bottom left)** gray matter active voxels, and **(bottom right)** white matter active voxels from simulations comparing the ability of Gaussian and constrained smoothing to recover cortical regions of interest in the presence of random white noise. The simulation was repeated for each of the 68 cortical regions of interest from the FreeSurfer parcellation maps. Dice coefficient and activation metrics are based on a percentile threshold where the total number of active voxels is equal to the size of the region of interest. Constrained smoothing demonstrated greater correlation to the ground truth across all FWHM values and better Dice scores at FWHM values greater than 3 mm (*p* < 0.001). Constrained smoothing more accurately concentrated the active voxels to the gray matter and decreased the number of active white matter voxels across all FWHM values as compared to Gaussian smoothing (*p* < 0.001).

As shown in Figure 4, constrained smoothing demonstrated higher accuracy as compared to Gaussian smoothing in terms of both DSC score at FWHM values greater than 3 mm and Pearson correlation across all FWHM values (*p* < 0.001). Further, as compared to Gaussian smoothing, constrained smoothing more accurately concentrated the positive predictions to the gray matter while decreasing the positive predictions in the white matter (*p* < 0.001). Additional simulations were conducted to validate the estimation of FWHM as well as to better understand the effect of voxel size on constrained smoothing outputs. These simulations demonstrated that Equation 1 accurately estimated the smoothing kernel width applied to both unconstrained and constrained graphs (see Supplementary Figure S13) and that smaller voxel sizes facilitated the effectiveness of constrained smoothing (see Supplementary Table S2).

### 3.2 Activation drift

**Figure 5:**
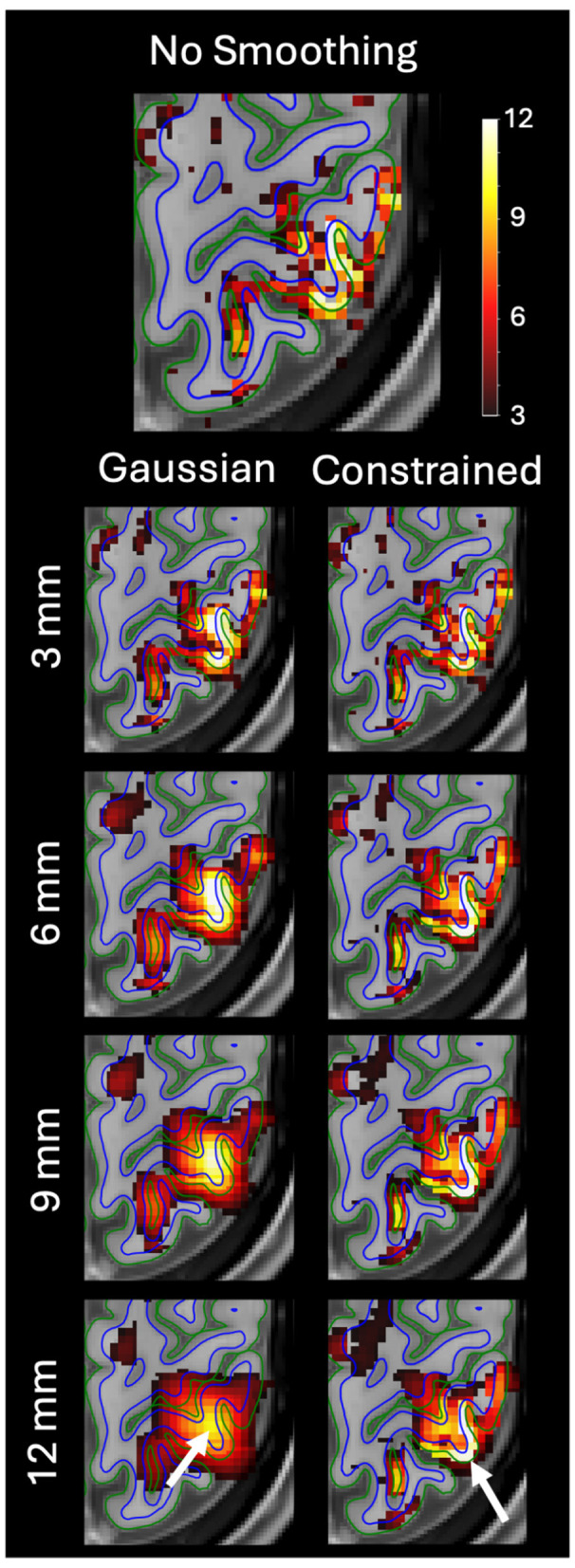
Example of the effect of Gaussian vs. anatomically constrained smoothing of the fMRI data on left-hand sensory task activation z-statistic maps generated by FSL FEAT. The task activation map without smoothing is shown on top. Results after smoothing with four different smoothing kernel widths (FWHM) are shown: 3, 6, 9, and 12 mm. While the differences were subtle at lower FWHM values, this example shows Gaussian smoothing spreading an activation cluster across the gray matter to white matter interface. This moves the center of the activation from the cortex into adjacent white matter. By contrast, the constrained smoothing method retains the peak level of activation within the cortical gray matter. The white arrows show the area with the Gaussian smoothing artifact.

Figure 5 shows an example of Gaussian and anatomically constrained smoothing from a single run for a given subject. Gaussian smoothing smoothed the signal across the white matter boundaries, creating smoothing artifacts in the white matter and moving the peak of the activation from the gray matter into the white matter. In contrast, by not smoothing across the cortical boundaries, constrained smoothing retains the peak activation within the gray matter of the gyrus.

**Figure 6:**
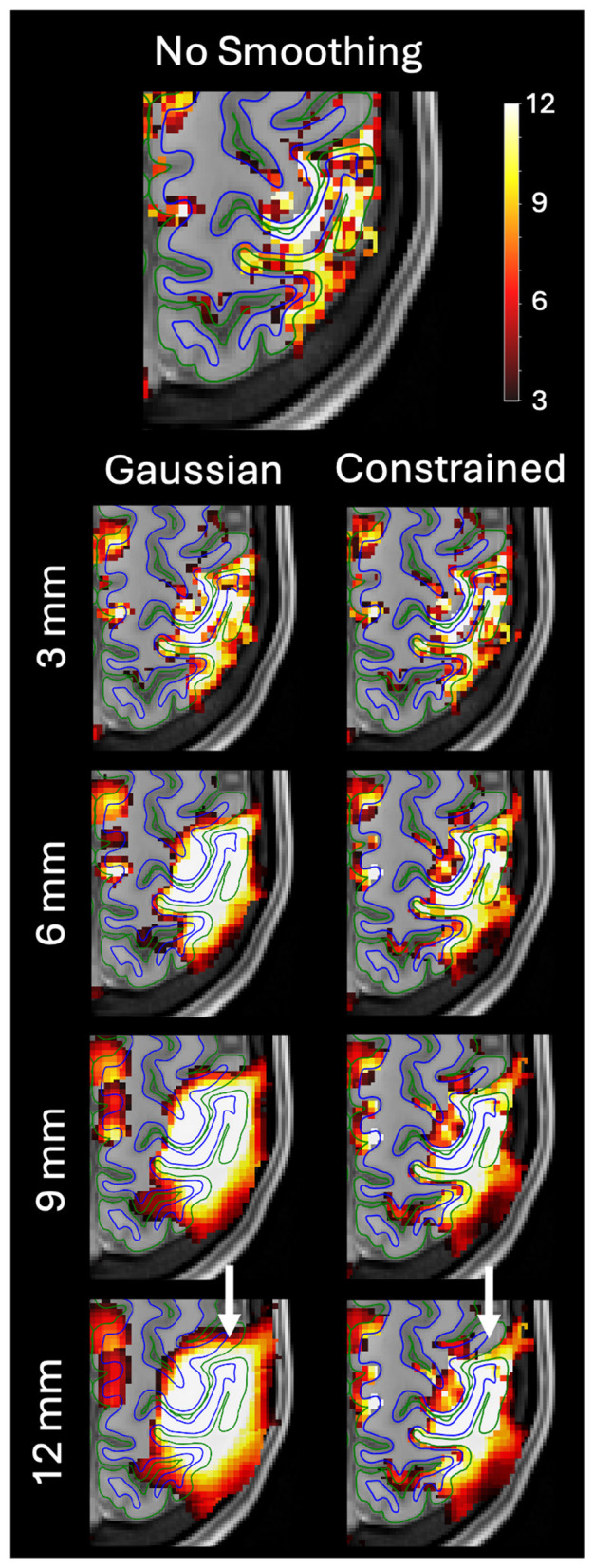
Example of the effect of Gaussian vs. anatomically constrained smoothing of the fMRI data on left-hand sensory task activation z-statistic maps generated by FSL FEAT. The task activation map without smoothing is shown on top. Results after smoothing with four different smoothing kernel widths (FWHM) are shown: 3, 6, 9, and 12 mm. While the differences were subtle at lower FWHM values, this example shows Gaussian smoothing spreading an activation cluster across a sulcus and into a separate gyrus that showed little to no activation without smoothing. By contrast, the constrained smoothing method does not smooth across the sulcus and the activation cluster is retained within the original area of activation. The white arrows show the area with the Gaussian smoothing artifact.

Figure 6 shows another example of Gaussian and anatomically constrained smoothing from a single run for a different subject. In this example, the Gaussian kernel smoothed the signal across the pial surface causing the activation cluster to spread into a neighboring gyrus. By contrast, constrained smoothing does not smooth the signal across the pial boundaries, which prevented the activation from spreading across the sulcus and into the neighboring gyrus. These examples highlight the ability of constrained smoothing to avoid common smoothing artifacts by preventing signal from being smoothed across the cortical boundaries.

**Figure 7:**
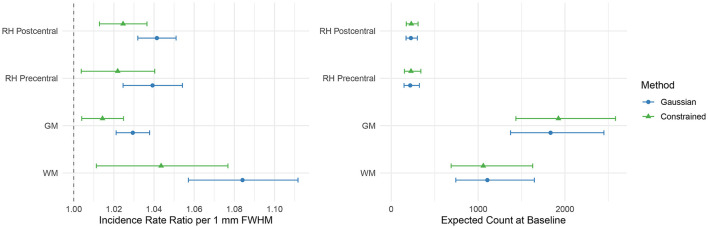
Effects of smoothing on the number of active voxels in the left-hand sensory stimulation task. **(Left)** Estimated incidence rate ratios and **(Right)** the intercepts representing the number of active voxels at baseline with 95% confidence intervals from the GLMMs examining the effects of Gaussian (blue) and constrained (green) smoothing on the number of active voxels. Relative to constrained smoothing, increasing Gaussian smoothing kernel widths increased the number of active voxels across the postcentral and gray matter (GM) regions of interest (*p* < 0.05). Increasing Gaussian and constrained smoothing kernel widths increased the number of active voxels across all regions (*p* < 0.05).

**Figure 8:**
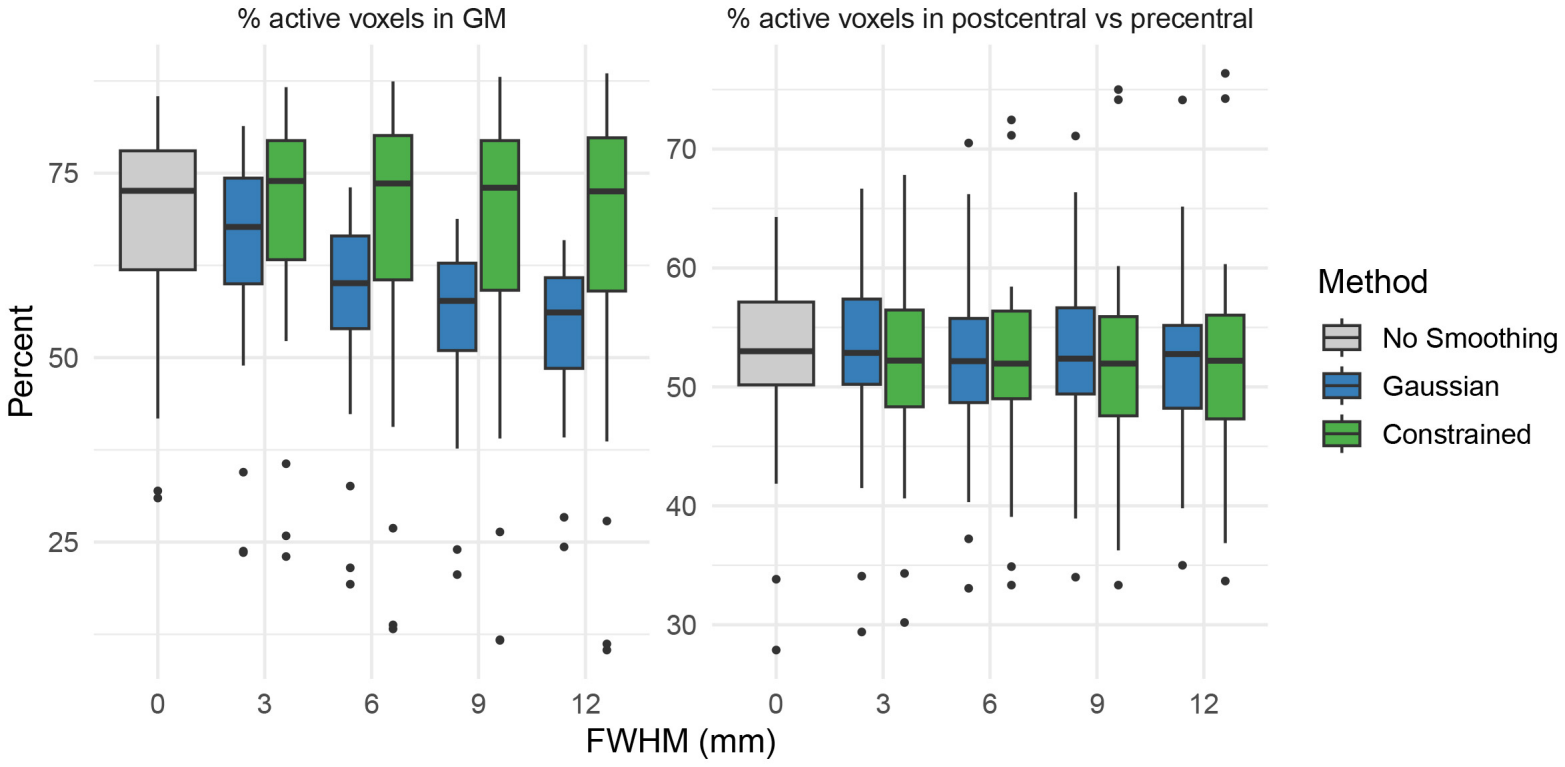
**(Left)** Percentage of active voxels in gray matter (GM) among all active voxels in gray and white matter; **(Right)** percentage in the postcentral gyrus among all active voxels in the precentral and postcentral gyri, across smoothing kernel widths for Gaussian and constrained smoothing. Relative to constrained smoothing, Gaussian smoothing showed a lower proportion of active voxels in gray matter across all smoothing levels (*p* < 0.001). There was no difference between the smoothing methods in the proportion of active voxels in the postcentral gyrus among all the voxels in the postcentral and precentral gyri (*p* > 0.05).

Figure 7 shows the slopes and intercepts for the GLMM models fit to the activation voxel counts for the sensory task activation dataset. Based on the fit GLMM models, increasing smoothing kernel widths increased the number of active voxels across all regions for both Gaussian and constrained smoothing (*p* < 0.05). Relative to constrained smoothing, increasing Gaussian smoothing increased the number of active voxels across the postcentral and gray matter (GM) regions of interest (*p* < 0.05). However, as shown in Figure 8, when evaluating the percentage of active voxels in the gray matter as compared to the total number of active voxels in the gray and white matter, constrained smoothing demonstrated an increased proportion of active gray matter voxels relative to Gaussian smoothing across all smoothing kernel widths (*p* < 0.001). When looking at the percentage of active voxels in the postcentral gyrus (the expected region of activation) as compared to the total number of active voxels in the postcentral and precentral gyri, there was no significant difference in the proportion of active postcentral voxels (*p* > 0.05). This indicates that constrained smoothing better retains the activation within the gray matter of the brain than Gaussian smoothing, but that we did not detect a significant difference in spreading of activation from the postcentral gyrus to the precentral gyrus between the methods.

### 3.3 Activation reliability

**Figure 9:**
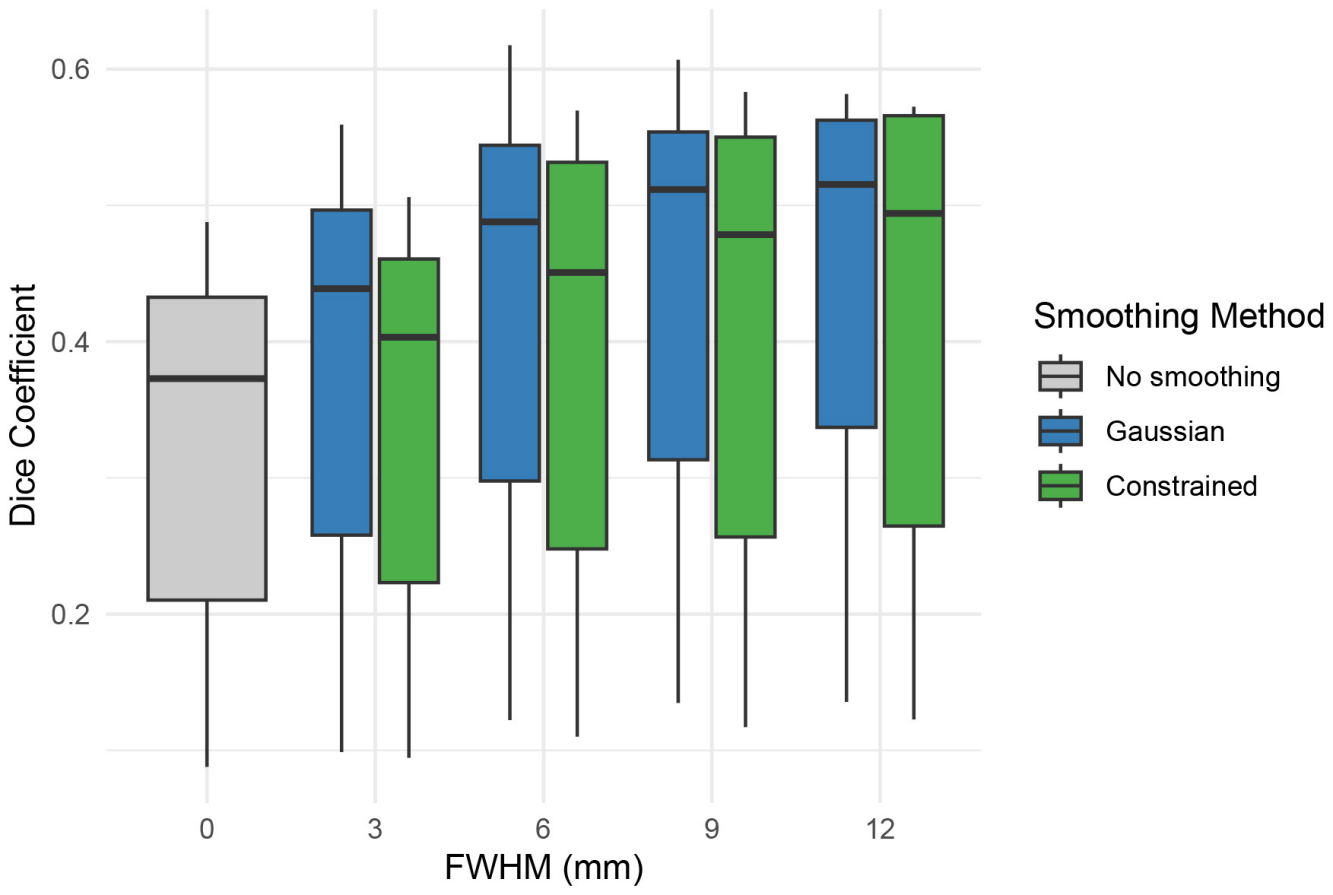
Effects of smoothing on task activation reliability with no smoothing (gray), Gaussian (blue), and our proposed constrained smoothing (green) as measured by Dice similarity coefficient at different smoothing kernel widths as measured by FWHM. Both smoothing methods demonstrated an increase in reliability as compared to no smoothing.

Increasing smoothing kernel widths for both constrained and Gaussian smoothing demonstrated increased reliability as measured by DSC (*p* < 0.001). The difference between the smoothing methods in terms of either the slope or the intercept of the GLMM was not statistically significant (*p* > 0.05). See Supplementary Figure S9 and Supplementary Table S6 for the detailed results of the GLMM model analyzing the effects of smoothing on the reliability as measured by DSC. Figure 9 shows the distributions of DSC at different smoothing kernel widths. When comparing the reliability at each smoothing interval using paired *t*-tests, the difference between the smoothing methods was not statistically significant (p > 0.05). These results indicate that both Gaussian and constrained smoothing are effective at increasing the reliability of task activation maps.

### 3.4 Activation accuracy

**Figure 10:**
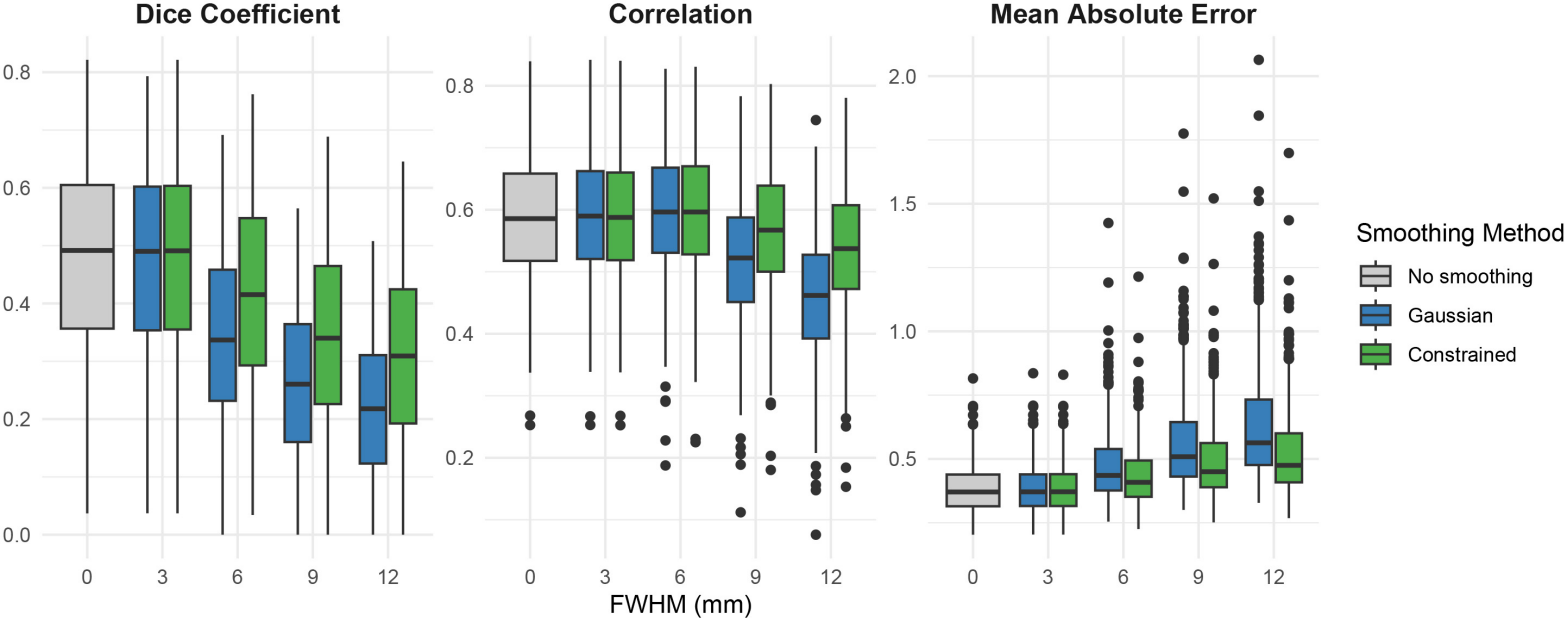
Effects of smoothing on task activation accuracy for motor activation compared to the 20-run average maps as the pseudo-ground truth. Single session motor task activation Dice similarity coefficient, Pearson correlation, and mean absolute error with no smoothing (gray), Gaussian (blue), and our proposed constrained smoothing (green) when evaluated against the 10 session precision functional maps from the MSC dataset.

As shown in Figure 10 and Table 1, Gaussian and constrained smoothing had varying effects on the accuracy of the resulting motor task activation maps depending on the metric utilized to compare to the 20-run average pseudo-ground truth maps. Overall, constrained smoothing increased the accuracy across all metrics relative to Gaussian smoothing when smoothing kernel width (as measured by FWHM) was greater than 3 mm. Relative to Gaussian smoothing, constrained smoothing showed increased DSC scores and Pearson correlations and decreased errors at FWHM values ≥ 6 mm, but decreased DSC scores and increased errors at FWHM = 3 mm. These results indicate that the constrained smoothing was able to effectively decrease the adverse effects of smoothing when greater smoothing kernel widths were applied. When comparing the methods to no smoothing, both Gaussian and constrained smoothing increased Pearson correlations to the pseudo-ground truth at FWHM values ≤ 6 mm. However, both smoothing methods also decreased Pearson correlations at FWHM values ≥ 9 mm, decreased DSC scores at FWHM values ≥ 6 mm, and increased mean absolute errors across all FWHM values.

**Table 1:** Pairwise statistical tests comparing smoothing methods across different FWHM values for motor activation accuracy relative to the 20-run average maps as the pseudo-ground truth.

Metric	FWHM	Comparison		Statistics		Significance
		Group 1	Group 2	*t*-statistic	*p*-value	
**Dice**	3	Gaussian	Constrained	-1.26	0.209	ns
	3	Gaussian	No Smoothing	-2.41	0.016	ns^†^
	3	Constrained	No Smoothing	-1.98	0.048	ns^†^
	6	Gaussian	Constrained	-29.62	< 0.001	^***^
	6	Gaussian	No Smoothing	-28.96	< 0.001	^***^
	6	Constrained	No Smoothing	-19.07	< 0.001	^***^
	9	Gaussian	Constrained	-29.77	< 0.001	^***^
	9	Gaussian	No Smoothing	-40.51	< 0.001	^***^
	9	Constrained	No Smoothing	-29.89	< 0.001	^***^
	12	Gaussian	Constrained	-26.90	< 0.001	^***^
	12	Gaussian	No Smoothing	-46.18	< 0.001	^***^
	12	Constrained	No Smoothing	-35.43	< 0.001	^***^
**Pearson r**	3	Gaussian	Constrained	36.83	< 0.001	^***^
	3	Gaussian	No Smoothing	58.42	< 0.001	^***^
	3	Constrained	No Smoothing	22.82	< 0.001	^***^
	6	Gaussian	Constrained	-4.89	< 0.001	^***^
	6	Gaussian	No Smoothing	3.12	0.002	^*^
	6	Constrained	No Smoothing	10.58	< 0.001	^***^
	9	Gaussian	Constrained	-53.90	< 0.001	^***^
	9	Gaussian	No Smoothing	-38.93	< 0.001	^***^
	9	Constrained	No Smoothing	-20.30	< 0.001	^***^
	12	Gaussian	Constrained	-77.88	< 0.001	^***^
	12	Gaussian	No Smoothing	-60.20	< 0.001	^***^
	12	Constrained	No Smoothing	-36.91	< 0.001	^***^
**MAE**	3	Gaussian	Constrained	-3.32	< 0.001	*
	3	Gaussian	No Smoothing	9.88	< 0.001	^***^
	3	Constrained	No Smoothing	14.83	< 0.001	^***^
	6	Gaussian	Constrained	24.38	< 0.001	^***^
	6	Gaussian	No Smoothing	27.58	< 0.001	^***^
	6	Constrained	No Smoothing	28.43	< 0.001	^***^
	9	Gaussian	Constrained	33.67	< 0.001	^***^
	9	Gaussian	No Smoothing	31.76	< 0.001	^***^
	9	Constrained	No Smoothing	28.92	< 0.001	^***^
	12	Gaussian	Constrained	38.01	< 0.001	^***^
	12	Gaussian	No Smoothing	33.46	< 0.001	^***^
	12	Constrained	No Smoothing	29.22	< 0.001	^***^

^†^Not significant after multiple comparison correction.

^*^p < 0.05,

^***^p < 0.001.

### 3.5 False positive voxels

**Figure 11:**
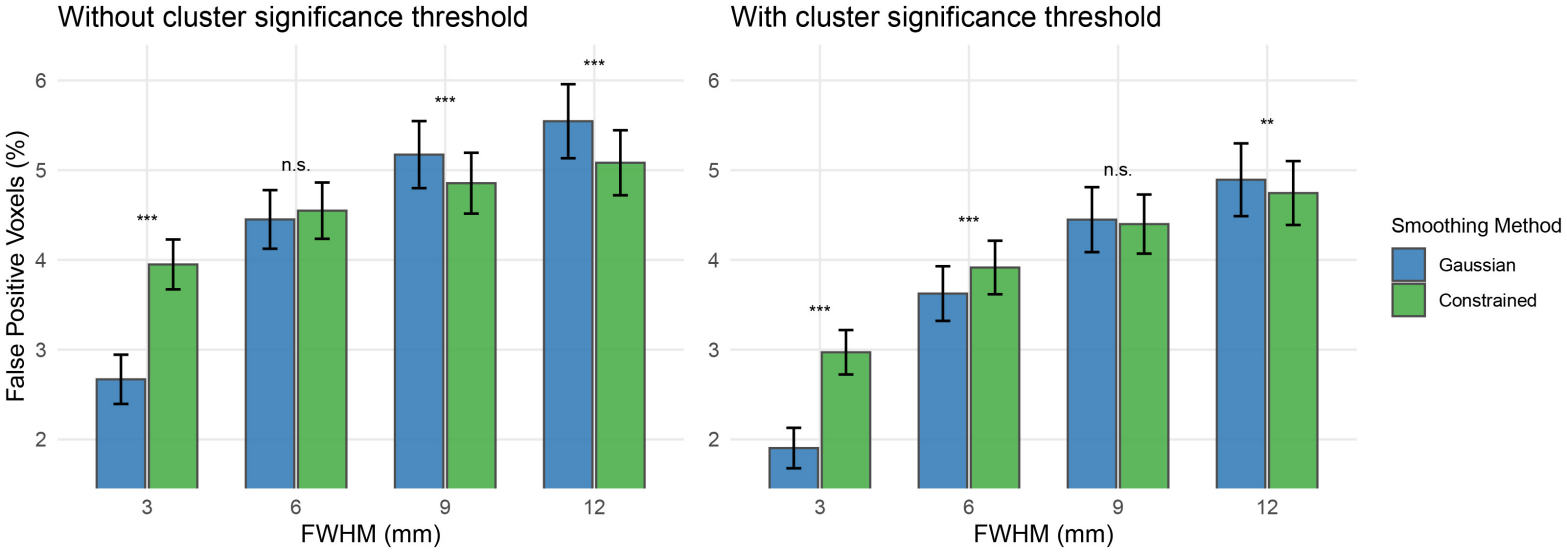
Average percentage of brain voxels classified as false positives from task analysis of resting state data. 140 subjects from the HCPA dataset with a combined 549 runs of resting state data were analyzed using FSL FEAT and a 30-second block design. This analysis was completed for both constrained and Gaussian smoothing with smoothing kernel widths of FWHM = 3, 6, 9, and 12 mm. The average percentage of voxels without cluster significance thresholding **(left)** and with FSL FEAT's cluster threshold method **(right)** are plotted. Constrained smoothing demonstrated a greater false positive voxel percentage when FWHM = 3 mm without cluster significance thresholding and when FWHM ≤ 6 mm with cluster significance thresholding (*p* < 0.001). Gaussian smoothing demonstrated a greater false positive voxel percentage when FWHM ≥ 9 mm without cluster significance thresholding (*p* < 0.001) and when FWHM = 12 mm with cluster significance thresholding (p < 0.01).

We conducted an analysis on the effects of constrained smoothing on the percentage of false positive voxels using 549 resting state scans from 140 subjects in the HCPA dataset. Each resting state scan was smoothed with Gaussian and constrained smoothing at FWHM values of 3, 6, 9, and 12 mm. The smoothed data was then analyzed with FSL FEAT using a task design with blocks lasting 30 seconds. The active voxels for each scan were determined both with and without using FSL FEAT's cluster significance threshold of p = 0.05. For each scan, smoothing method, and FWHM value, the number of active voxels as a percentage of total brain voxels was computed and considered the per-scan false positive percentage. As shown in Figure 11, the average false positive percentage tended to increase with increasing smoothing kernel width, with the greatest false positive voxel percentage resulting from Gaussian smoothing with FWHM = 12 mm. Constrained smoothing demonstrated increased per-scan false positive percentage at FWHM = 3 mm without cluster significance thresholding and at FWHM ≤ 6 mm with cluster significance thresholding (*p* < 0.001). Gaussian smoothing demonstrated increased per-scan false positive percentage at FWHM ≥ 9 mm without cluster significance thresholding (*p* < 0.001) and at FWHM = 12 mm with cluster significance thresholding (*p* < 0.01). When cluster significance thresholding was used, neither method demonstrated a mean false positive voxel percentage greater than 5%. Qualitatively, no substantial differences in the spatial organization of false positive voxels were detected when examining the average false positive percentage maps (see Supplementary Figures S2, S3).

### 3.6 Connectivity

**Figure 12:**
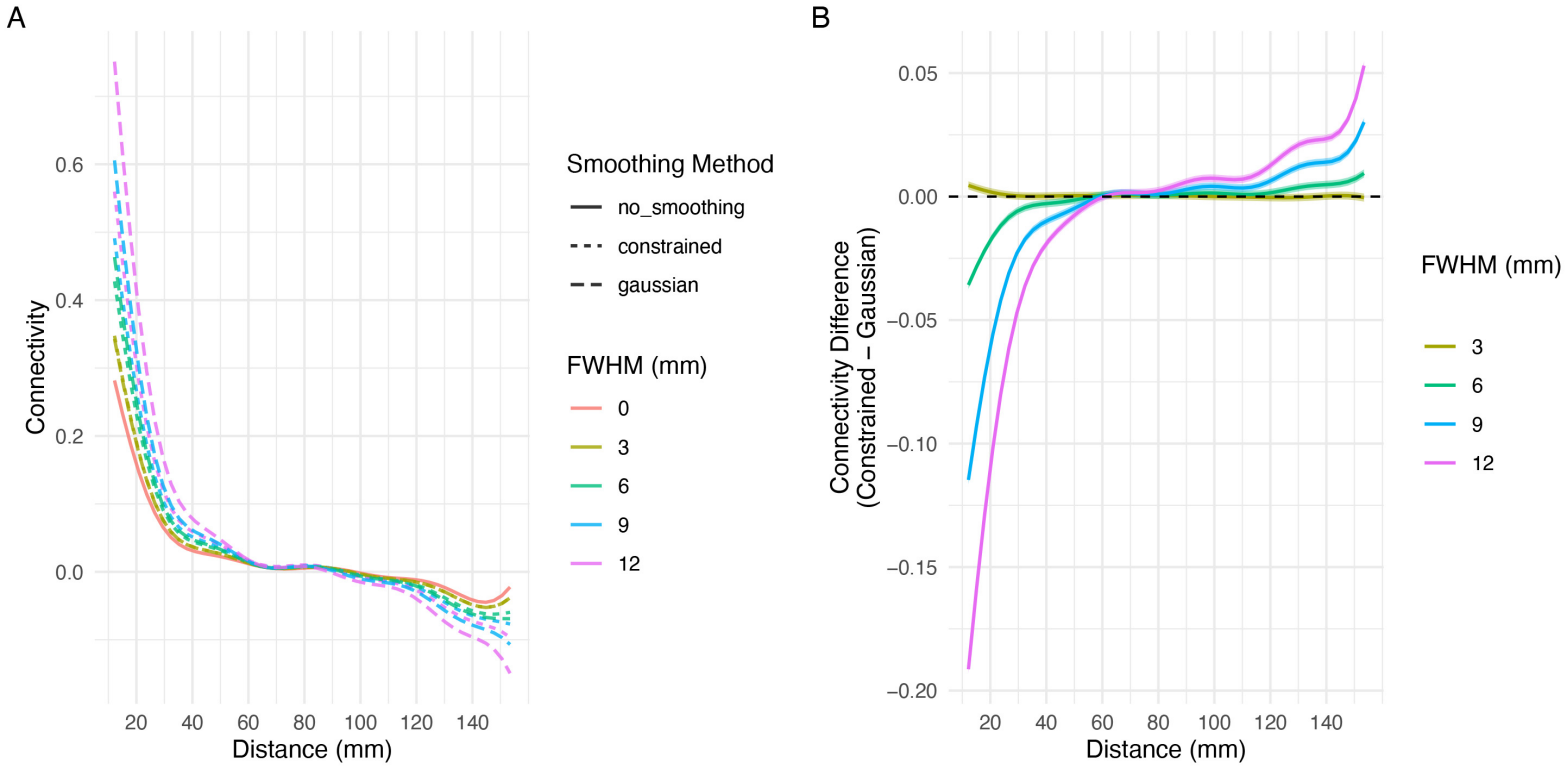
Effect of smoothing on the relationship between region-to-region distance and resting state functional connectivity. **(A)** The relationship between the Euclidean distance between ROI centroids and functional connectivity as measured by the Fisher z-transformed Pearson correlation coefficients. The graph shows that ROI pairs that are closer together ( ≤ 60 mm) tend to have positive functional connectivity and that this relationship is exacerbated by smoothing. **(B)** The difference between the connectivity (with 95% confidence intervals) between constrained and Gaussian smoothing. Constrained smoothing demonstrated less inflation of connectivity at FWHM ≥ 6 mm as compared to Gaussian smoothing.

Figure 12 shows the predicted resting state functional connectivity values based on the distance between ROI centroids under each smoothing condition after fitting the GAMM described by Equation 3. The GAMM was fitted based on the resting state functional connectivity data of 100 low-motion subjects from the Human Connectome Project in Aging. As shown in Figure 12A, functional connectivity was positive when the distance between ROIs was less than 60 mm. Increasing smoothing kernel widths (as defined by FWHM in mm) of both Gaussian and constrained smoothing further increased the functional connectivity between spatially proximal ROIs. As shown in Figure 12B, at FWHM values of 6 mm or greater, Gaussian smoothing demonstrated more inflation of functional connectivity values for close ROI pairs. Figure 12 also shows that ROI pairs greater than 110 mm apart have a slight negative correlation, and that this negative correlation is also inflated by greater smoothing kernel widths.

**Figure 13:**
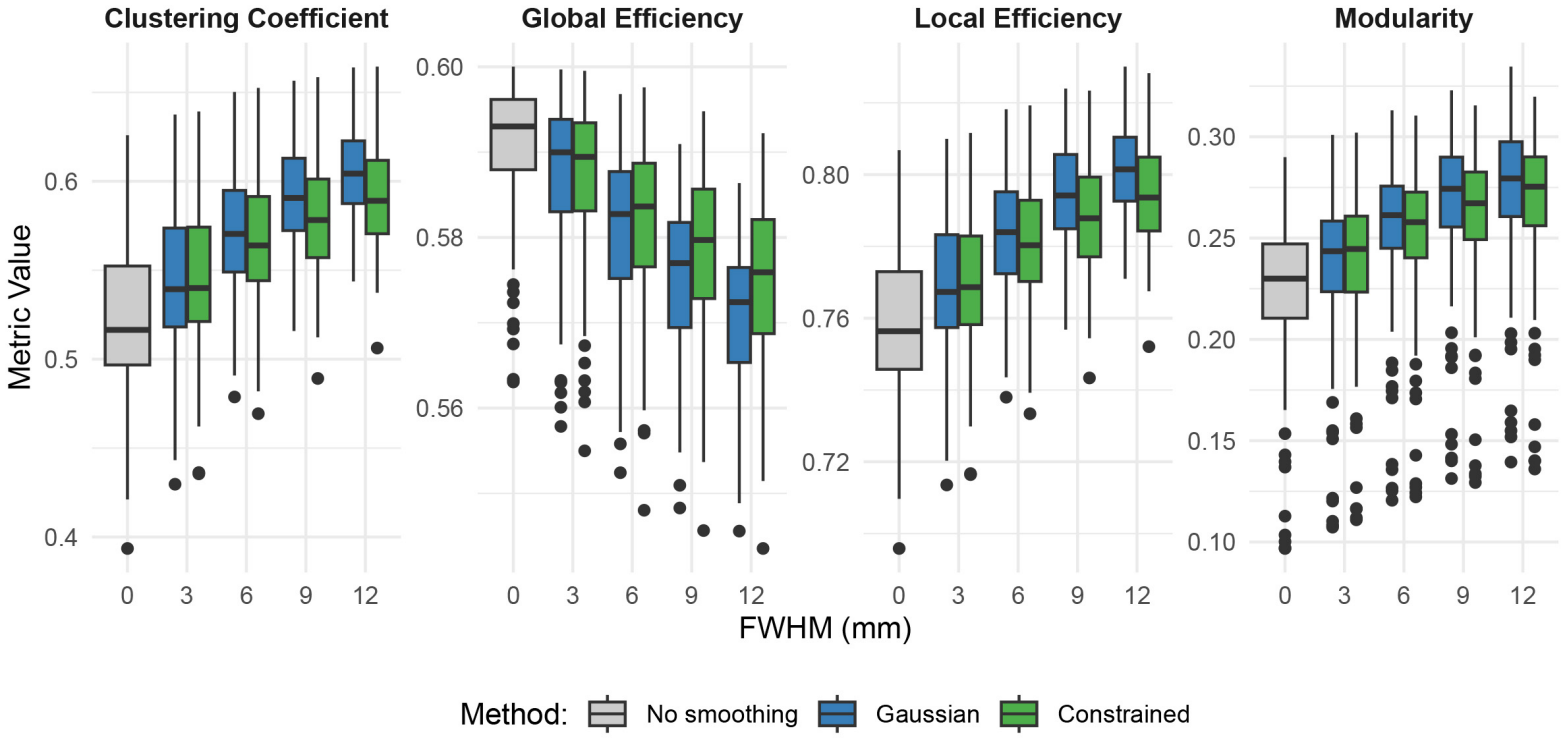
Effects of smoothing on resting state functional connectivity graph theory metrics. Smoothing (both constrained and Gaussian) increased clustering coefficient, local efficiency, and modularity metrics while decreasing global efficiency.

Figure 13 shows the effects of both constrained and Gaussian smoothing on functional connectivity derived graph theory metrics from the same 100 subjects from the HCPA dataset. Both Gaussian and constrained smoothing significantly altered the graph theory metrics under all smoothing kernel widths (*p* < 0.001 at FWHM ≥ 3 mm for local efficiency and clustering coefficient, and *p* < 0.05 at FWHM = 3 mm along with *p* < 0.001 at FWHM ≥ 6 mm for modularity and global efficiency). Relative to Gaussian smoothing, constrained smoothing had less of an effect on the local efficiency, global efficiency, and clustering coefficient at higher smoothing kernel widths of FWHM ≥ 9 mm (*p* < 0.05). Gaussian and constrained smoothing showed no differences at any smoothing kernel width when looking at modularity (*p* >0.05).

## 4 Discussion

This study demonstrated that unconstrained Gaussian smoothing can create smoothing artifacts by smoothing signal across anatomical boundaries. Anatomically constrained volumetric smoothing was shown to avoid smoothing artifacts by preventing smoothing kernels from crossing the white and pial surfaces of the cortex. When smoothing images with simulated cortical signal in the presence of white noise, constrained smoothing limited the spreading of signal to within well-defined anatomical boundaries. When larger smoothing kernels (FWHM > 3 mm) were used, Gaussian smoothing moved the cortical signal into the white matter and decreased the accuracy of the resulting image. In contrast, constrained smoothing kept the signal confined to the cortex, resulting in greater accuracy than Gaussian smoothing. Under a sensory task condition, both Gaussian and constrained smoothing increased the reliability across repeated acquisitions as compared to no smoothing. However, Gaussian smoothing tended to decrease the proportion of active voxels located within the gray matter relative to the white matter. When comparing the percentage of false positive voxels between smoothing methods and kernel widths, the mean percentage of false positive voxels remained below 5% for both methods across the tested kernel widths with cluster significance thresholding. During resting state connectivity analysis, unconstrained Gaussian smoothing artificially inflated the functional connectivity between nearby regions of interest. Relative to Gaussian smoothing, constrained smoothing had less of an effect on the functional connectivity between nearby regions at larger smoothing kernel widths. Together, these results indicate that constrained volumetric smoothing offers an alternative to Gaussian smoothing that avoids some of the smoothing artifacts associated with Gaussian smoothing.

Interestingly, while smoothing did increase the reliability of fMRI activations, it did not increase the accuracy of activations across all metrics when measured against the 20-run mean activation maps. DSC scores were the highest and errors the lowest when no smoothing was applied. However, both Gaussian and constrained smoothing did improve the accuracy in terms of Pearson r correlation values at shorter smoothing kernel widths. Therefore, the distribution of the parameters at low smoothing widths became more correlated to the target pseudo-ground truth parameters despite the shifts in the magnitudes. When comparing the smoothing methods' ability to recover the cortical regions in the presence of simulated white noise, constrained smoothing performed much better than Gaussian smoothing. Therefore, the poor accuracy in the real-fMRI results could be due to a noisy definition of ground truth, too large a voxel size (4 mm isotropic) for constrained smoothing to smooth effectively, or other factors involved in the analysis process. Future research could further evaluate under which conditions constrained smoothing is most advantageous.

An important caveat to the results of this study is that constrained smoothing is inherently more conservative than unconstrained Gaussian smoothing. Constrained smoothing inherently aggregates fewer data points than unconstrained smoothing at identical smoothing kernel widths, which could partially explain why constrained smoothing limits some of the negative effects observed with large Gaussian smoothing kernels. In unconstrained smoothing, the number of data points being aggregated during a smoothing depends solely on the smoothing kernel width. However, constrained smoothing restricts the connections such that the number of data points being aggregated is always less than in the unconstrained case. Similar to how a 1D smoothing kernel applies less smoothing than a 2D smoothing kernel of the same nominal width, constrained smoothing non-symmetrically lowers the effective dimensionality of the smoothing operation, even when the same nominal smoothing kernel width is used. Therefore, when constraining the smoothing to not aggregate data across cortical boundaries, we are also lowering the effective level of smoothing being applied.

Consequently, for constrained smoothing, the effective level of smoothing depends on the connectivity of the pruned graph. A more connected graph allows for data to be aggregated between more data points. When constraining the graph using the cortical surfaces, the connectivity of the graph is dependent upon the voxel size of the image. Larger voxel sizes will result in fewer connections, particularly in the gray matter, where the voxel width might be similar to the cortical thickness. Conversely, smaller voxels allow for more connectivity and, thus, more data aggregation. We demonstrated the effect of graph connectivity and voxel size on the effective smoothing level in Supplementary Section 4.2. We also tested the effect of artificially increasing the resolution of the fMRI data through upsampling to a 1 mm grid before applying constrained smoothing. Overall, we noticed that the additional resampling steps increased the baseline smoothness of the resulting images across all smoothing kernel widths, but it was difficult to determine if this method produced better results (see Supplementary Section 3 for more details). However, since smaller voxels produce better connectivity, it is likely that constrained smoothing could be most advantageous in high resolution fMRI experiments.

A related interpretive limitation of this current work is that the voxel size varied between studies. The voxel size was 0.8 mm isotropic for the simulated data, 1.7 mm x 1.7 mm x 4 mm for the sensory fMRI data, 4 mm isotropic for the motor fMRI data, and 2 mm isotropic for the resting state fMRI data. For both Gaussian and constrained smoothing, the effective level of data aggregation for a given smoothing kernel width depends on the voxel dimensions of the underlying data. Therefore, the smoothing kernel widths common to all studies (FWHM = 3, 6, 9, and 12 mm) represent different levels of data aggregation for each of the experiments. For example, smoothing with a kernel width of 3 mm produces less data aggregation when smoothing data with a 4 mm isotropic voxel size than when smoothing data with a 0.8 mm isotropic voxel size. For this reason, comparison of the results across the experiments should be done with caution. To facilitate comparison of smoothing kernel widths between experiments, Supplementary Table S8 reports the smoothing kernel widths relative to the voxel dimensions for each dataset.

One surprising result was that volumetric smoothing (both Gaussian and constrained) significantly changed graph theory metrics in resting state functional connectivity analysis. Any differences between constrained and Gaussian smoothing were marginal as compared to the overall effect of smoothing on the graph theory metrics. Interestingly, a previous study found that smoothing did not have a significant effect on the graph theory network connectivity measures (Candemir, 2023). The difference between our results and those from (Candemir 2023) could be due to the large difference in the number of ROIs considered. We used the Schaefer/Yeo atlas with 1,000 parcels (Schaefer et al., 2017) while the previous study by Candemir used only 32 ROIs (Candemir, 2023). Therefore, it is likely that using fewer, larger ROIs may make resting state connectivity analysis using graph theory metrics less sensitive to smoothing artifacts.

Surface-based signal smoothing has been previously considered as the primary way to avoid volumetric smoothing artifacts in fMRI data (Coalson et al., 2018). However, constrained smoothing may be an alternative to surface-based signal smoothing methods as it likewise is able to prevent volumetric smoothing artifacts. Volumetric constrained smoothing has the additional advantage of smoothing fMRI data in the volume itself rather than requiring the data to be transformed into a cortical surface-based representation of the data. While useful in many studies, surface-based approaches move the data out of the voxel space, which has been the standard space for acquiring, processing, analyzing, and reporting of fMRI studies for decades. Because of their novelty, surface-based approaches require both new tools and modification of standard tools to allow researchers to perform data processing and analysis. While constrained volumetric smoothing does still require reconstructed surfaces for processing, it could appeal to researchers desiring to process and analyze their data in volumetric rather than surface space. Further, constrained smoothing could be adapted to selectively smooth fMRI data in areas of the brain beyond the cortex, such as along reconstructed tracts within the white matter (Abramian et al., 2021).

Like surface-based approaches, our proposed approach mitigates some smoothing artifacts resulting from Gaussian smoothing by preventing smoothing kernels from crossing cortical boundaries. Similar to our results, previous studies have shown that surface-based smoothing avoided the artifacts injected by Gaussian-based approaches (Brodoehl et al., 2020; Coalson et al., 2018). However, surface-constrained volumetric smoothing does not inherit all the benefits of surface-based approaches. One benefit of surface-based approaches is the better alignment of subject data in the template space (Ghosh et al., 2010; Coalson et al., 2018). A 2018 study by Coalson et al. demonstrated that surface-based registration better aligned distinct cortical regions than volumetric registration (Coalson et al., 2018). Surface-based analytical approaches also consolidate the BOLD data along the vector normal to the cortical surfaces (Khan et al., 2011). In theory, this approach takes into account that the different layers of the cortex work together to perform a given task, and, therefore, their BOLD values can be combined, assuming the data was acquired with a resolution greater than the thickness of the cortex. Additionally, surface-based approaches allow for enhanced visualization of the data and results (Coalson et al., 2018). However, future advancements in the registration and signal aggregation of volumetric data could mitigate the current limitations of volume-based approaches.

One hurdle to the adoption of our proposed constrained volumetric smoothing implementation is that it requires the fMRI data to be in the same space as the surfaces, which is the T1w space in fmriprep. Group activation studies would then need to be transformed into the template space for further analysis. We found that this transformation into MNI space took much longer than running the actual constrained smoothing operation, 17 minutes for the transformation as compared to 2.5 minutes for running the constrained smoothing. A technical consideration regarding our implementation is that the ray tracing occasionally failed to prune all the connections crossing the cortical boundaries, resulting in an atypical number of large connected components. We were able to check the number of connected components during the pruning process and correct by eliminating edges that were close to intersecting cortical surfaces and by slightly adjusting the direction of the connections before rerunning the ray tracing step. If the correct number of connected components are still not identified after these steps, then the code produces an error. This ensures that the outputs are not produced when the program suspects that the graph is not adequately pruned.

An alternative approach to volumetric constrained smoothing may be to perform smoothing in the surface space and then project the results back to the cortex using the Connectome Workbench (Marcus et al., 2013). This approach may have an advantage in reduced computational load due to smoothing only over about 60,000 vertices as compared to smoothing over millions of voxels. However, we could not find this approach to volumetric smoothing demonstrated in any published literature. Similarly, (Blazejewska et al. 2019), proposed a hybrid 2D/3D smoothing approach that applies an intracortical smoothing kernel to the layers of the cortex which they demonstrated to be beneficial in small-voxel 7 T fMRI studies (Blazejewska et al., 2019).

Future work could be done to use this graph methodology for smoothing small-voxel fMRI (Blazejewska et al., 2019), Bayesian inference of fMRI activation (Oskarsson et al., 2022; Mejia et al., 2020; Sidén et al., 2021), and to separately smooth structures such as subcortical regions (Glasser et al. 2013) or cortical layers (Blazejewska et al., 2019). Bayesian inference could be of particular interest as Bayesian techniques can determine the optimal level of smoothing from the data using a spatial prior (Penny et al., 2005). Therefore, providing additional information about the anatomical structure of the brain through a constrained graph for Bayesian inference could result in more accurate activation modeling (Mejia et al., 2020; Abramian et al., 2020).

Future studies should take into account that surface-constrained volumetric smoothing likely produces non-stationary and anisotropic spatial smoothness across the brain, which can violate the Gaussian random field assumptions typically used by parametric models (e.g., FSL FEAT) for computing voxel- and cluster-level statistics (Eklund et al., 2016). Therefore, activation maps generated using parametric models following constrained smoothing, as was done in this study, may not satisfy the assumptions required for valid voxel- and cluster-level statistical inference. In addition, the sensory and motor analyses did not use a multiple-comparison correction procedure. Those activation maps should therefore be for comparative purposes, rather than as evidence for the precise spatial localization of statistically significant activation. Accordingly, the sensory and motor results may not generalize to typical fMRI analysis pipelines in which multiple-comparison correction is standard practice. Future research should use nonparametric permutation testing to perform valid voxel- and cluster-level statistical inference on volumetric data smoothed with constrained smoothing approaches (Winkler et al., 2014).

## 5 Conclusion

This study showed that unconstrained Gaussian smoothing spreads activation across cortical boundaries, increases white matter activation, and biases graph theory connectivity metrics. Anatomically constrained smoothing mitigated these artifacts by preventing smoothing across cortical boundaries, while still increasing reliability. These results support anatomically constrained volumetric smoothing as a practical alternative to unconstrained smoothing, and motivate future work integrating constrained smoothing with nonparametric statistical inference.

## Data Availability

The sensory task fMRI data analyzed in this study can be found at openneuro.org under dataset ds005009 version 1.0.0. The fMRI data for the Midnight Scan Club analyzed in this study can be found at openneuro.org under dataset ds000224 version 1.0.4. Data analyzed in this study from the Human Connectome Project in Aging can be accessed through the National Institute of Mental Health Data Archive at nda.nih.gov. Data collection for the Human Connectome Project in Aging was supported by the National Institute on Aging of the National Institutes of Health under Award Number U01AG052564.
