# Supplementary material for: Anatomically constrained volumetric smoothing enhances fMRI reliability while avoiding smoothing artifacts
Source: Front Neuroimaging. 2026 May 1;5:1753534. doi: 10.3389/fnimg.2026.1753534 (PMC13175814; doi:10.3389/fnimg.2026.1753534)
Supplement: Supplementary file 1 [file Data_Sheet_1.pdf]

Supplementary Material

1 MODELING THE NUMBER OF ACTIVE VOXELS

In the sensory task activation analysis, the number of active voxels was modeled using a GLMM with a negative binomial distribution and a log link function. This decision was based on the distributions of the number of active voxels per region as shown in Figure S1.

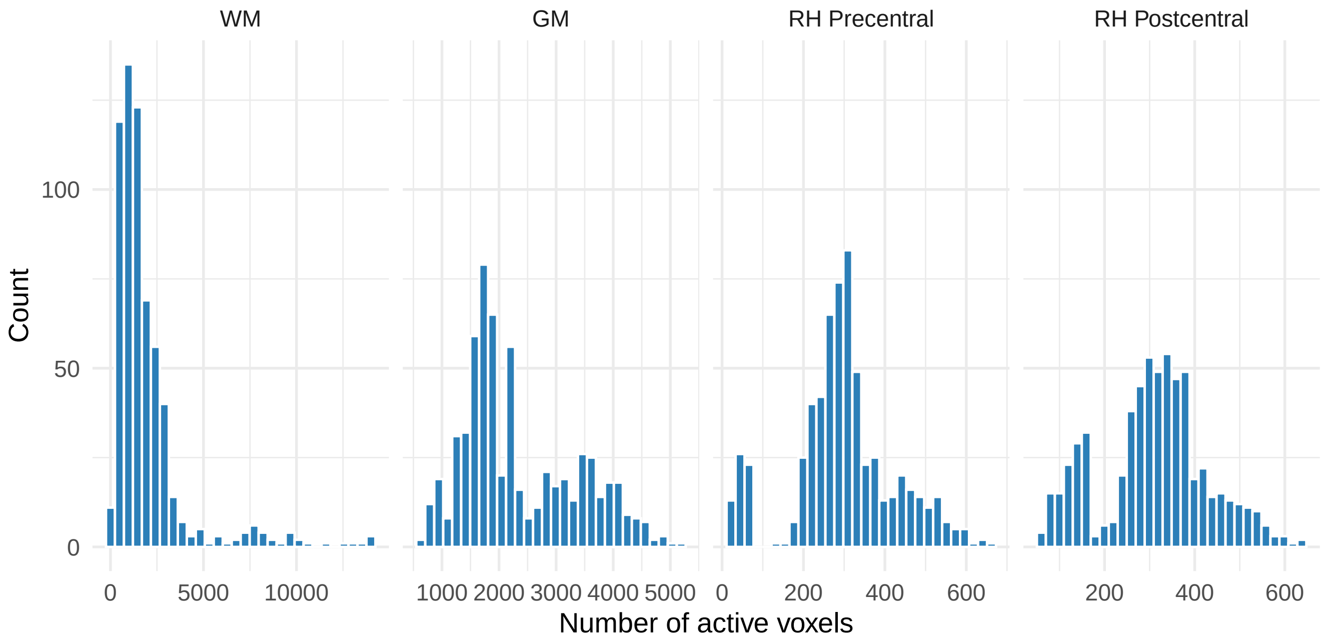

Figure S1: Histogram plots showing the number of active voxels for all smoothing levels and methods (including no smoothing) for each region of interest: white matter (WM), gray matter (GM), right precentral (RH Precentral), and right postcentral (RH Postcentral).

2 FALSE POSITIVE VOXELS

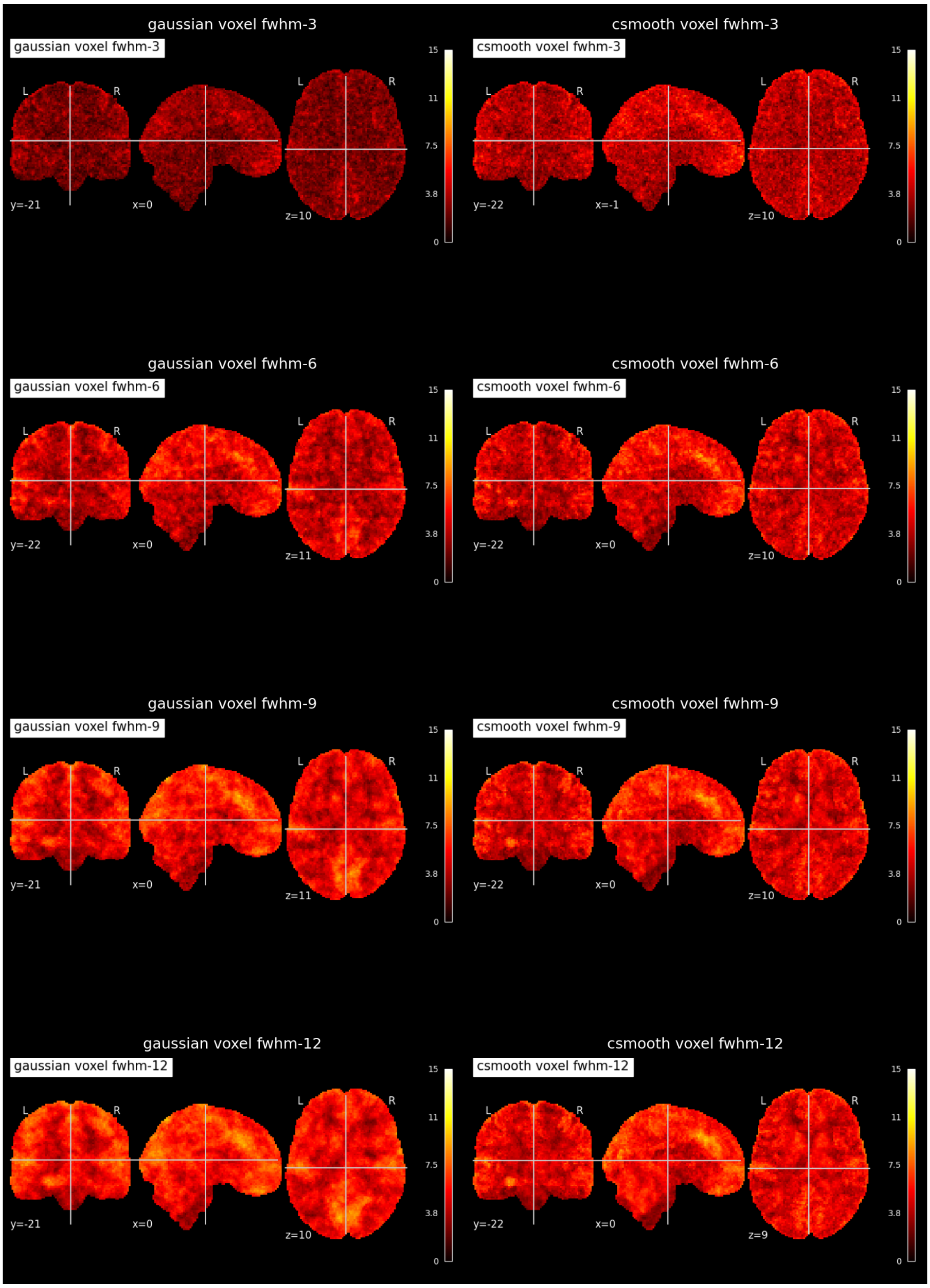

Figure S2: Voxelwise false positive percentage maps for a task analysis performed on resting state data with a voxelwise threshold, z-statistic $>$ 3.1 without cluster significance correction, used to determine active voxels (i.e., false positive voxels). The plotted voxelwise false positive percentage maps show the percentage of runs in which a given voxel was active.

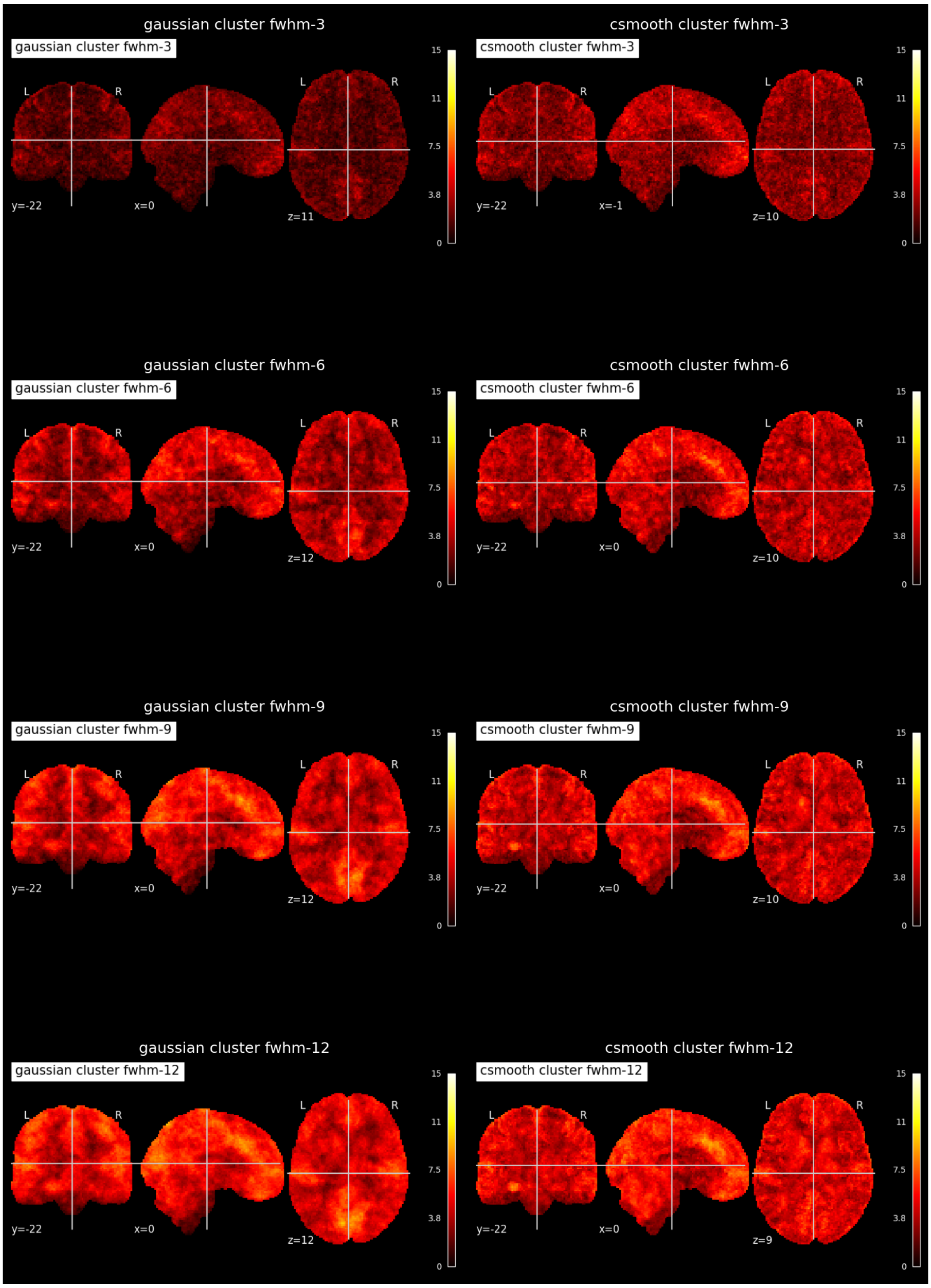

Figure S3: Voxelwise false positive percentage maps for a task analysis performed on resting state data with a voxelwise threshold, z-statistic $>$ 3.1, and FSL FEAT's cluster significance threshold of p = 0.05 used to determine active voxels (i.e., false positive voxels). The plotted voxelwise false positive percentage maps show the percentage of runs in which a given voxel was active.

3 CONSTRAINED SMOOTHING WITH RESAMPLING

In addition to the main analysis, we conducted an analysis in which the images were resampled to a 1 mm isotropic resolution. This extra upsampling step allowed for more connections in the graph and could, in theory, allow for more effective smoothing. Under this alternative approach, the constrained graph was built and pruned with voxel sizes of 1 mm. The fMRI images were upsampled to the 1 mm isotropic resolution and constrained smoothing was performed. After smoothing, the fMRI images were resampled back into the original resolution. While this allowed for more connections in the graph, the resampling of the fMRI images also likely adversely affected the outcome metrics for the constrained smoothing approach, particularly at baseline. The results with this extra resampling step are shown below alongside the results without resampling as a reference. Constrained smoothing with resampling markedly increased the computational requirements due to the cubic increase in the number of voxels being considered. We required approximately 60 GB of memory to run constrained smoothing with resampling as compared to 10 GB without resampling, and smoothing a single fMRI file took approximately 15 minutes as compared to 2.5 minutes without resampling.

3.1 Sensory activation example images

As shown in Figure S4 and Figure S5, constrained smoothing with resampling may increase white matter activation relative to constrained smoothing without resampling. However, these examples also show that the primary activation area appears to remain in the cortex despite a slight increase in white matter activation. Further, constrained smoothing with resampling still avoids spreading activation across a gyrus, as shown in Figure S5.

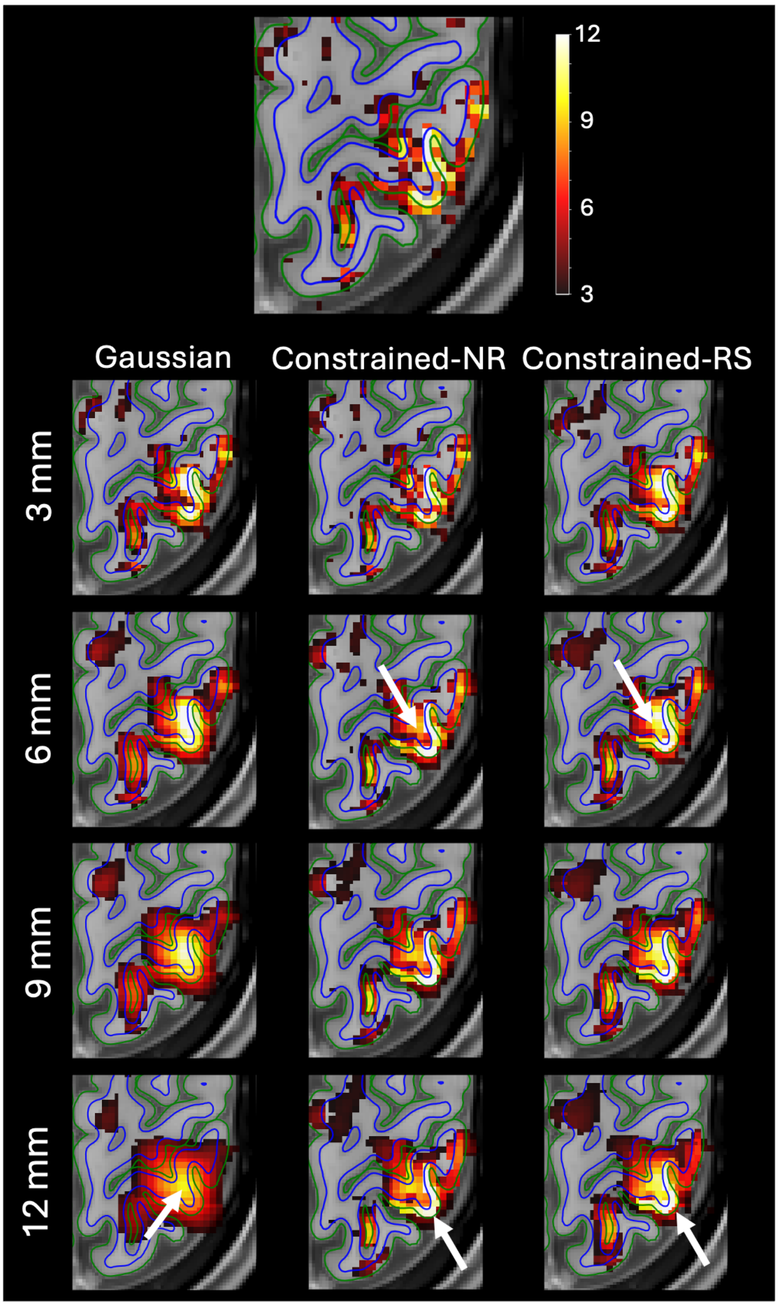

Figure S4: Example of the effect of Gaussian smoothing as compared to anatomically constrained smoothing both with no resampling (Constrained-NR) and with resampling (Constrained-RS) of the fMRI data on left-hand sensory task activation z-statistic maps generated by FSL FEAT. The task activation map without smoothing is shown on top. The example shows four different smoothing kernel widths (FWHM): 3, 6, 9, and 12 mm. Compared to constrained smoothing without resampling, constrained smoothing with resampling demonstrated increased white matter activation, as highlighted by the white arrows on the FWHM of 6 mm row. However, both constrained smoothing methods retained the majority of activation within the gray matter, while Gaussian smoothing shifted the activation into the white matter, as highlighted by the arrows on the FWHM = 12 mm row.

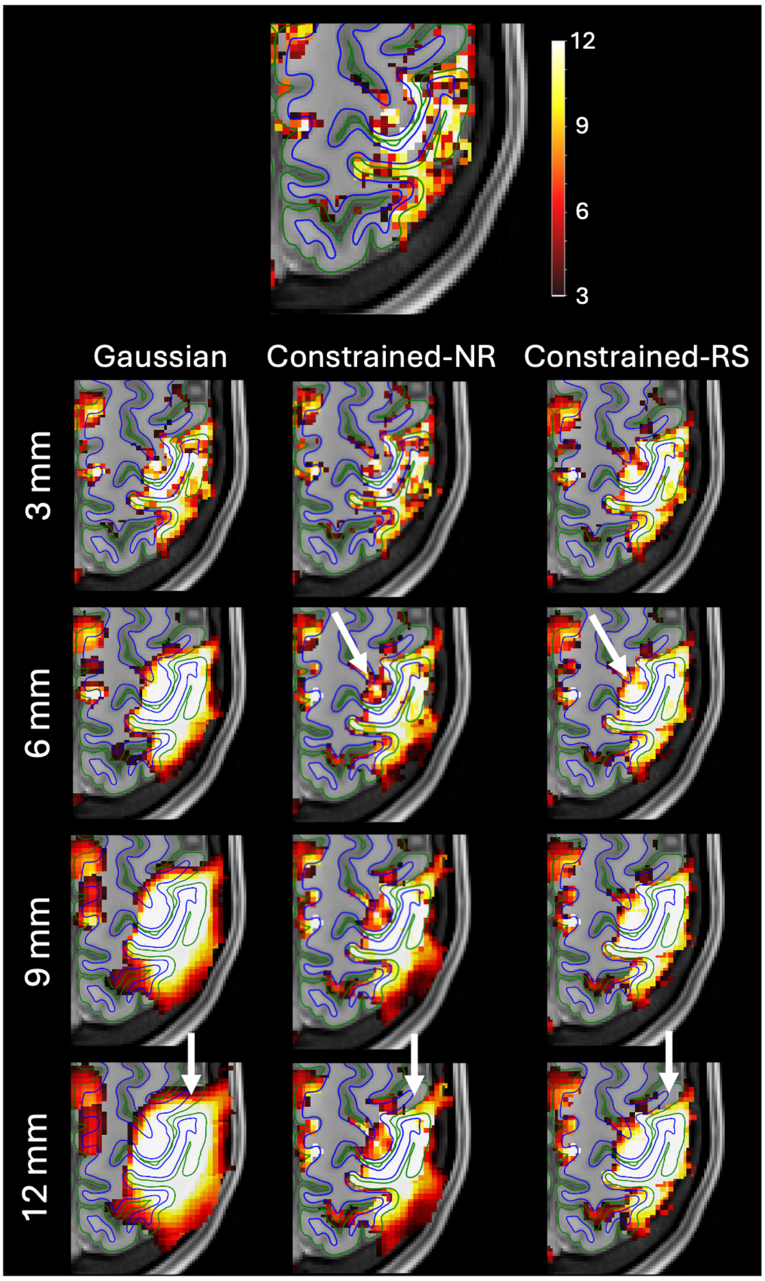

Figure S5: Example of the effect of Gaussian smoothing as compared to anatomically constrained smoothing both with no resampling (Constrained-NR) and with resampling (Constrained-RS) of the fMRI data on left-hand sensory task activation z-statistic maps generated by FSL FEAT. The task activation map without smoothing is shown on top. The example shows four different smoothing kernel widths (FWHM): 3, 6, 9, and 12 mm. Compared to constrained smoothing without resampling, constrained smoothing with resampling demonstrated increased white matter activation, as highlighted by the white arrows on the FWHM of 6 mm row. However, unlike Gaussian smoothing, both constrained smoothing methods prevented signal from spreading across a sulcus to a nearby gyrus, as highlighted by the arrows on the FWHM of 12 mm row.

3.2 Active-voxels analysis with resampling for sensory task

In the sensory task activation analysis on the active voxel counts, constrained smoothing with resampling increased the outcome metrics at baseline but produced smaller increases with increasing smoothing kernel widths. For example, as shown in Figure S6, the intercepts for the generalized linear mixed model (GLMM) on the number of active voxels for the constrained smoothing approach with resampling were higher, and the slopes were lower than for the other approaches.

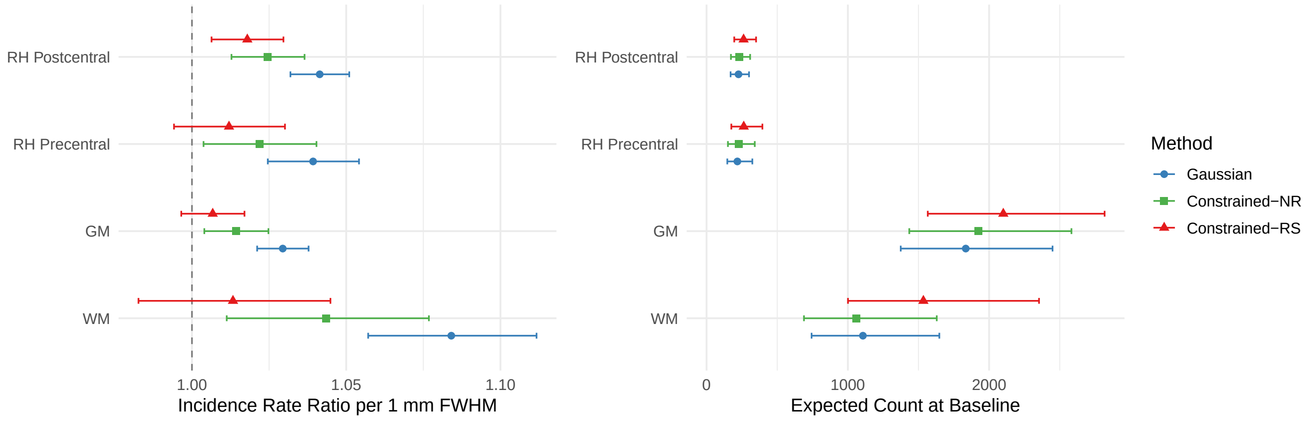

Figure S6: Parameter estimates for the slopes and intercepts with 95% confidence intervals of the fitted active voxel counts GLMM with Gaussian smoothing, constrained smoothing (Constrained-NR), and constrained smoothing with resampling (Constrained-RS) in the sensory task activation analysis.

Figure S7 shows the number of active voxels for each smoothing condition and smoothing kernel width, including no smoothing in the left-hand sensory task activation analysis.

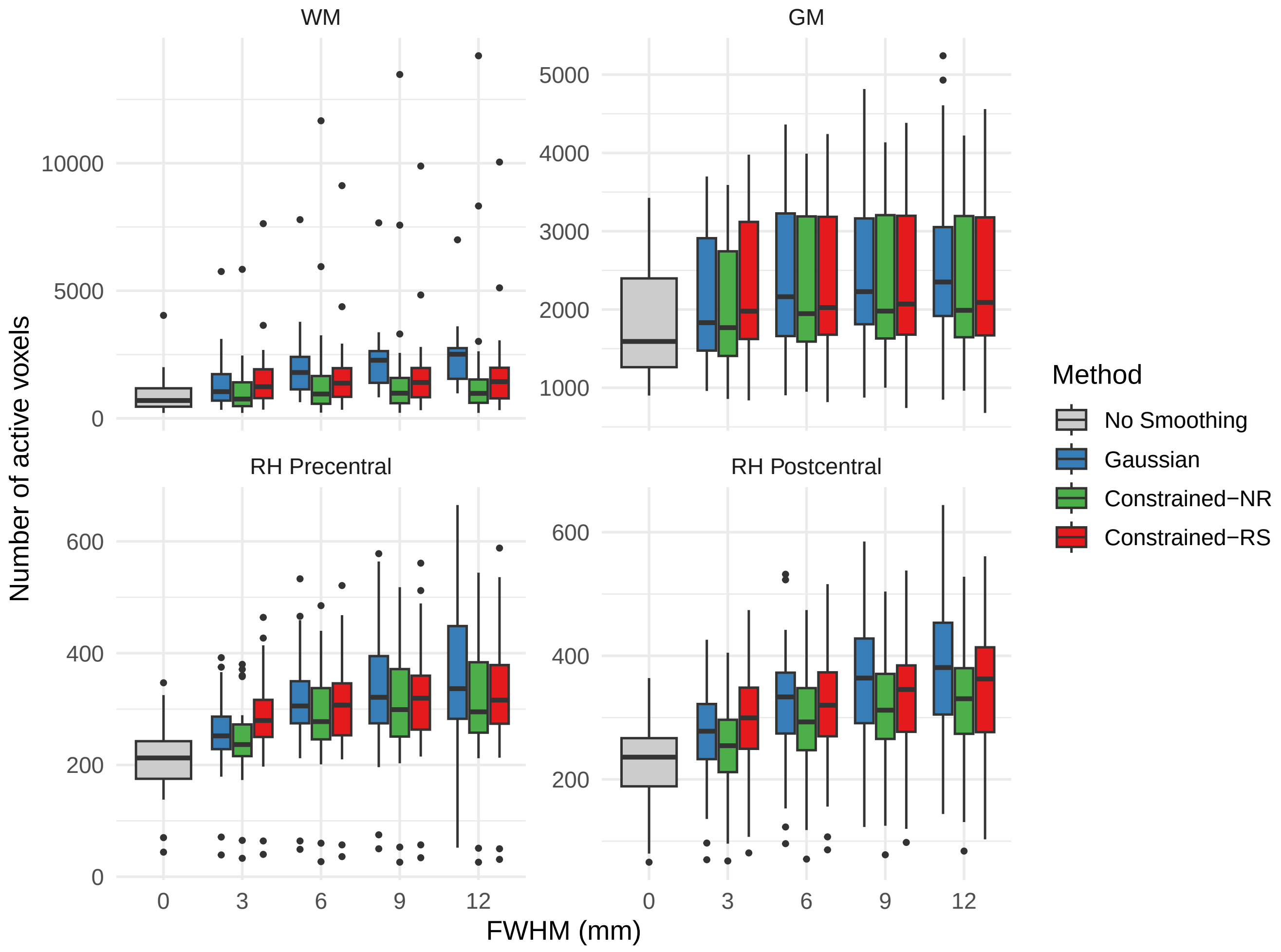

Figure S7: The number of active voxels for each region and smoothing method in the sensory task activation analysis.

3.3 Sensory activation reliability analysis with resampling

As shown in Figure S8, we also examined the reliability of the sensory task activation maps, comparing Gaussian smoothing, constrained smoothing without resampling (Constrained-NR), and constrained smoothing with resampling (Constrained-RS).

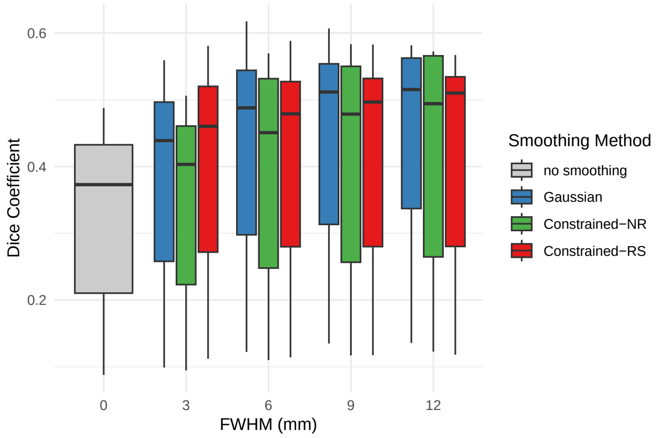

Figure S8: Sensory task activation reliability with Gaussian smoothing, constrained smoothing without resampling (Constrained-NR), and constrained smoothing with resampling (Constrained-RS).

The parameter estimates for the GLMM model examining the effect of smoothing on sensory task fMRI reliability as measured by Dice similarity coefficients of the activation maps are shown in Figure S9. The results are shown for both constrained smoothing without resampling (Constrained-NR) and constrained smoothing with resampling (Constrained-RS).

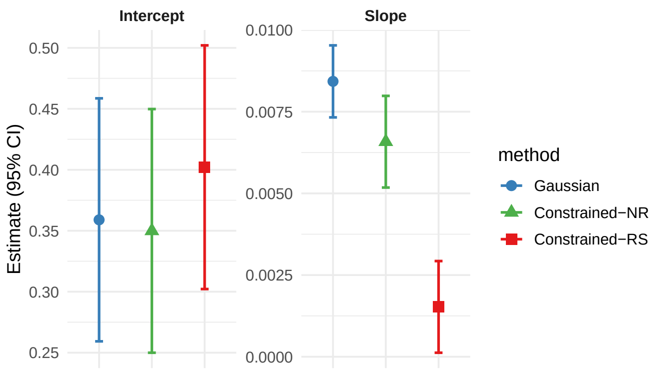

Figure S9: Parameter estimates and 95% confidence intervals for the GLMM model comparing the reliability as measured by Dice similarity coefficient of Gaussian, constrained smoothing (Constrained-NR), and constrained smoothing with resampling (Constrained-RS).

3.4 Motor task activation accuracy analysis with resampling

As shown in Figure S10, the accuracy of the motor task activation results (with the 20-run average maps as the pseudo-ground truth) was affected by whether constrained smoothing was performed with or without upsampling. Notably, constrained smoothing with resampling appears to have lower Dice similarity coefficients but higher Pearson correlations as compared to constrained smoothing without resampling.

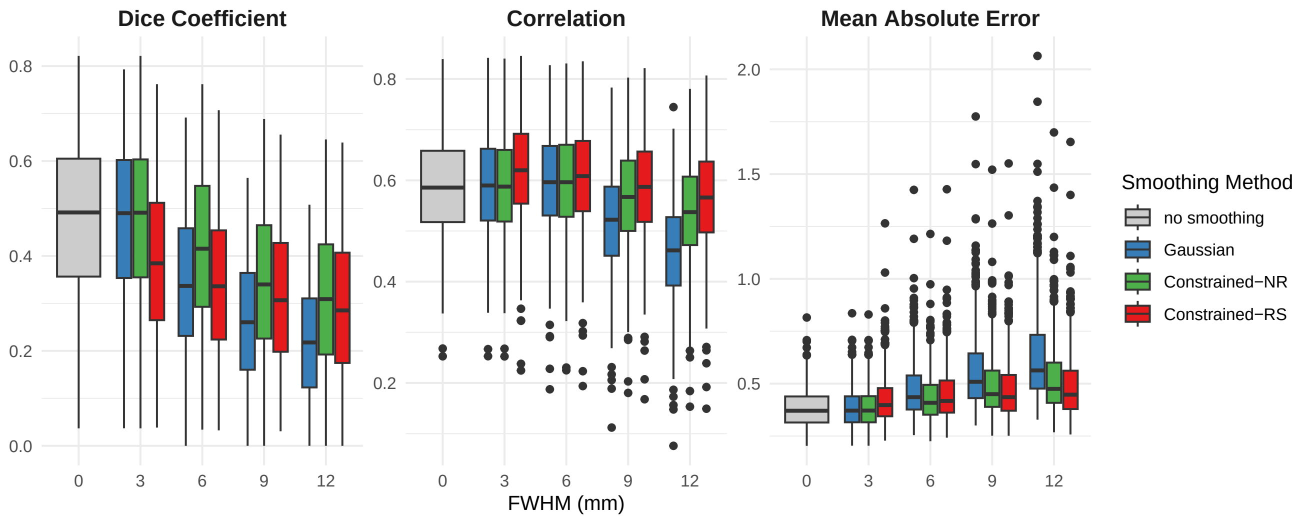

Figure S10: Motor task activation accuracy with Gaussian smoothing, constrained smoothing without resampling (Constrained-NR), and constrained smoothing with resampling (Constrained-RS).

Table S1 shows the paired t-tests comparing the accuracy of constrained smoothing with resampling to Gaussian and no smoothing.

Table S1. Pairwise statistical tests comparing constrained smoothing with resampling (Constrained-RS) to Gaussian smoothing and no smoothing across different FWHM levels for motor activation accuracy relative to the 20-run average maps as the pseudo-ground truth.

Metric	FWHM	Comparison		Statistics		Significance
		Group 1	Group 2	t-statistic	p-value	
Dice	3	Gaussian	Constrained-RS	22.85	<0.001	***
	3	Constrained-RS	No Smoothing	-22.17	<0.001	***
	6	Gaussian	Constrained-RS	-0.80	0.422	ns
	6	Constrained-RS	No Smoothing	-28.38	<0.001	***
	9	Gaussian	Constrained-RS	-23.73	<0.001	***
	9	Constrained-RS	No Smoothing	-33.30	<0.001	***
	12	Gaussian	Constrained-RS	-23.69	<0.001	***
	12	Constrained-RS	No Smoothing	-36.22	<0.001	***
Pearson r	3	Gaussian	Constrained-RS	-28.51	<0.001	***
	3	Constrained-RS	No Smoothing	30.91	<0.001	***
	6	Gaussian	Constrained-RS	-35.46	<0.001	***
	6	Constrained-RS	No Smoothing	10.44	<0.001	***
	9	Gaussian	Constrained-RS	-100.07	<0.001	***
	9	Constrained-RS	No Smoothing	-5.53	<0.001	***
	12	Gaussian	Constrained-RS	-114.10	<0.001	***
	12	Constrained-RS	No Smoothing	-16.83	<0.001	***
MAE	3	Gaussian	Constrained-RS	-19.03	<0.001	***
	3	Constrained-RS	No Smoothing	18.84	<0.001	***
	6	Gaussian	Constrained-RS	42.18	<0.001	***
	6	Constrained-RS	No Smoothing	21.39	<0.001	***
	9	Gaussian	Constrained-RS	43.30	<0.001	***
	9	Constrained-RS	No Smoothing	22.70	<0.001	***
	12	Gaussian	Constrained-RS	42.88	<0.001	***
	12	Constrained-RS	No Smoothing	23.23	<0.001	***

†Not significant after multiple comparison correction (adjusted p = 0.197)

*p < 0.05, **p < 0.01, ***p < 0.001

3.5 Connectivity analysis with resampling

The connectivity analysis with resampling demonstrated an initial increase in outcome metrics when FWHM = 3 mm, but trends similar to the no-resampling approach at higher smoothing kernel widths. Figure S11 shows the predicted resting state functional connectivity values based on the distance between ROI centroids under each smoothing condition after fitting the GAMM described by Equation 3 in the main manuscript.

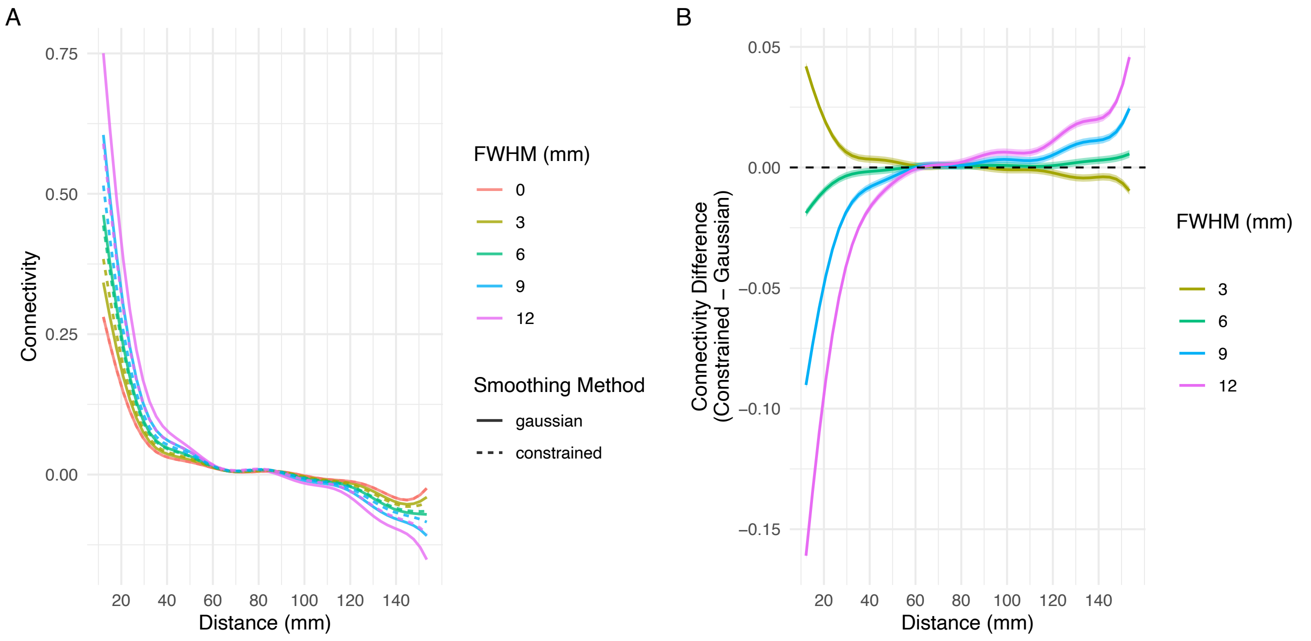

Figure S11: Effects of smoothing on the relationship between distance and resting state functional connectivity. (A) The relationship between the Euclidean distance between ROI centroids and functional connectivity as measured by the Fisher z-transformed Pearson correlation coefficients. The graph shows that ROI pairs that are closer together ($\leq$ 60 mm) tend to have positive functional connectivity and that this relationship is exacerbated by smoothing. (B) The difference between the connectivity (with 95% confidence intervals) between Gaussian smoothing and constrained smoothing with resampling. Constrained smoothing with resampling demonstrated more inflation of connectivity at FWHM $=$ 3 mm but less inflation of connectivity at FWHM $\geq$ 6 mm as compared to Gaussian smoothing.

Figure S12 shows the effects of both constrained smoothing with resampling and Gaussian smoothing on functional connectivity derived graph theory metrics from 100 subjects in the HCPA dataset.

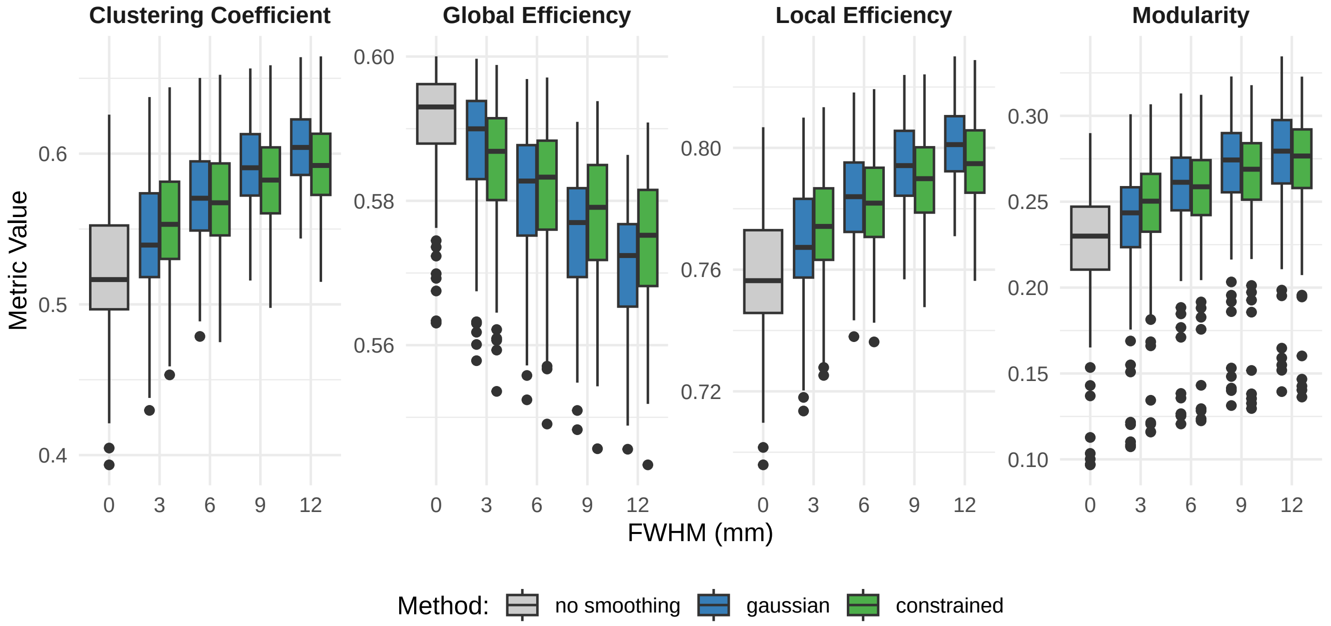

Figure S12: Effects of smoothing on resting state functional connectivity graph theory metrics. Smoothing (both constrained and Gaussian) increased clustering coefficient, local efficiency, and modularity metrics while decreasing global efficiency.

4 SIMULATIONS

In order to further inform and verify our conclusions regarding constrained smoothing, we conducted three sets of simulations. First, we sought to validate the estimation of FWHM by smoothing a noisy image with a known Gaussian kernel and then estimating the FWHM using the proposed equation. Second, we conducted an experiment to better understand the effect of voxel size on the constrained smoothing approach. Third, we created noisy images with a simulated cortical ground truth and compared Gaussian and constrained smoothing's ability to recover the ground truth.

4.1 FWHM validation

To validate that Equation 1 accurately estimates the FWHM of an applied Gaussian kernel, we simulated a 3D image with random white noise of mean zero and standard deviation of one and isotropic voxel size of 1 mm. We then applied Gaussian smoothing kernels separately to the noisy image ranging from 1 mm FWHM to 15 mm FWHM. After each separate smoothing, we used Equation 1 to estimate the smoothness of the data in terms of FWHM. The FWHM was estimated both on the unconstrained graph and the constrained graph by pruning the graph with surfaces from a subject in the MSC dataset. As shown in Figure S13, the estimation of the FWHM closely matches the actual smoothing kernel applied in both the unconstrained and the constrained graph.

4.2 Effect of voxel size on constrained smoothing

Based on our qualitative observations, we hypothesized that the voxel size of the graph affects the amount of smoothing applied to the underlying data by affecting the connectedness of the graph. To isolate the effect of the voxel size on the graph, we used the surfaces from a subject in the MSC to prune a graph at 1

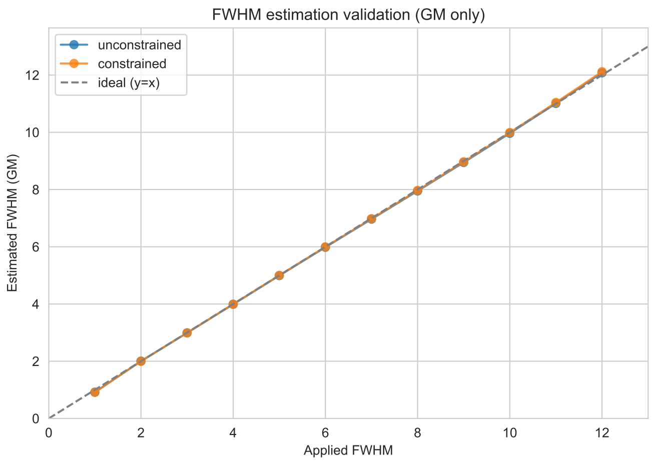

Figure S13: Estimated FWHM and the actual FWHM of a Gaussian smoothing kernel applied to a 3D image with random white noise. The estimated values are shown for the gray matter voxels only (the area of interest in most fMRI analyses), but results were similar for white matter voxels as well. The estimated FWHM values closely matched those of the applied smoothing kernel for the levels of smoothing investigated in this study.

mm isotropic voxel size and another at 3 mm isotropic voxel size. A second fully connected graph with 1 mm voxels was also created and then pruned according to the connections of the 3 mm voxel graph. In this pruning, each 1 mm voxel node was assigned to a corresponding parent node in the 3 mm graph. Only connections within nodes sharing the same parent and connections to nodes with connected parent nodes were allowed to remain. A random white noise image with mean of zero, and standard deviation of 1, and isotropic voxel size of 1 mm was then smoothed with an estimated FWHM of 6 mm as well as with a fixed tau of 13. The remaining standard deviation of the gray matter voxels after smoothing was then measured and compared against the smoothing runs.

Table S2. Effect of voxel size on constrained smoothing

Experiment	Graph	τ	GM STD	WM STD
FWHM = 6 mm	1 mm	13.5784	0.1513	0.1269
FWHM = 6 mm	3 mm	12.9154	0.3414	0.4460
τ = 13	1 mm	13.0000	0.1601	0.1319
τ = 13	3 mm	13.0000	0.3445	0.4452

As shown in Table S2, constrained smoothing with the 3 mm graph connections showed higher levels of remaining white noise in both the gray matter and white matter as compared to the constrained smoothing on the 1 mm graph. This indicates that the effective level of smoothing depends on the voxel size used to define the graph connections and not only on the choice of τ.

5 ADDITIONAL STATISTICS

This section contains detailed statistical tables for the results that were summarized in the main paper.

5.1 Simulation study

Table S3. Pairwise statistical tests comparing constrained smoothing (without resampling) to Gaussian smoothing and no smoothing across different FWHM levels for simulation the accuracy results shown in Figure 4.

metric	fwhm	group1	group2	t	p value	adjusted p value	significance
dice	3	gaussian	constrained	-2.27	0.026	0.473	ns
dice	3	gaussian	no smoothing	199.54	1.08E-94	1.95E-93	***
dice	3	constrained	no smoothing	102.87	1.77E-75	3.18E-74	***
dice	6	gaussian	constrained	-29.46	4.56E-40	8.21E-39	***
dice	6	gaussian	no smoothing	110.43	1.57E-77	2.83E-76	***
dice	6	constrained	no smoothing	249.51	3.47E-101	6.24E-100	***
dice	9	gaussian	constrained	-46.28	1.38E-52	2.48E-51	***
dice	9	gaussian	no smoothing	94.41	5.33E-73	9.59E-72	***
dice	9	constrained	no smoothing	327.86	3.99E-109	7.18E-108	***
dice	12	gaussian	constrained	-54.63	2.73E-57	4.91E-56	***
dice	12	gaussian	no smoothing	86.32	2.05E-70	3.69E-69	***
dice	12	constrained	no smoothing	339.18	4.11E-110	7.40E-109	***
pearson_r	3	gaussian	constrained	-8.19	1.10E-11	1.98E-10	***
pearson_r	3	gaussian	no smoothing	28.30	5.45E-39	9.80E-38	***
pearson_r	3	constrained	no smoothing	26.97	1.08E-37	1.94E-36	***
pearson_r	6	gaussian	constrained	-13.45	1.05E-20	1.89E-19	***
pearson_r	6	gaussian	no smoothing	47.79	1.70E-53	3.07E-52	***
pearson_r	6	constrained	no smoothing	38.59	1.71E-47	3.08E-46	***
pearson_r	9	gaussian	constrained	-23.57	3.80E-34	6.84E-33	***
pearson_r	9	gaussian	no smoothing	60.92	2.11E-60	3.79E-59	***
pearson_r	9	constrained	no smoothing	50.78	3.25E-55	5.84E-54	***
pearson_r	12	gaussian	constrained	-35.10	7.37E-45	1.33E-43	***
pearson_r	12	gaussian	no smoothing	63.47	1.42E-61	2.56E-60	***
pearson_r	12	constrained	no smoothing	60.99	1.96E-60	3.53E-59	***
active_wm	3	gaussian	constrained	11.19	5.60E-17	1.01E-15	***
active_wm	3	gaussian	no smoothing	-10.79	2.69E-16	4.85E-15	***
active_wm	3	constrained	no smoothing	-11.07	8.74E-17	1.57E-15	***
active_wm	6	gaussian	constrained	10.53	7.61E-16	1.37E-14	***
active_wm	6	gaussian	no smoothing	-10.02	5.92E-15	1.07E-13	***
active_wm	6	constrained	no smoothing	-11.19	5.44E-17	9.79E-16	***
active_wm	9	gaussian	constrained	10.08	4.57E-15	8.23E-14	***
active_wm	9	gaussian	no smoothing	-2.78	0.007	0.127	ns
active_wm	9	constrained	no smoothing	-11.20	5.24E-17	9.43E-16	***
active_wm	12	gaussian	constrained	9.77	1.64E-14	2.96E-13	***
active_wm	12	gaussian	no smoothing	2.18	0.032	0.584	ns
active_wm	12	constrained	no smoothing	-11.21	5.22E-17	9.39E-16	***
active_gm	3	gaussian	constrained	-6.46	1.37E-08	2.46E-07	***
active_gm	3	gaussian	no smoothing	11.14	6.75E-17	1.21E-15	***
active_gm	3	constrained	no smoothing	10.24	2.39E-15	4.30E-14	***
active_gm	6	gaussian	constrained	-9.57	3.63E-14	6.54E-13	***
active_gm	6	gaussian	no smoothing	11.58	1.21E-17	2.17E-16	***
active_gm	6	constrained	no smoothing	10.75	3.15E-16	5.67E-15	***
active_gm	9	gaussian	constrained	-9.85	1.16E-14	2.09E-13	***
active_gm	9	gaussian	no smoothing	12.26	8.98E-19	1.62E-17	***
active_gm	9	constrained	no smoothing	10.90	1.73E-16	3.11E-15	***
active_gm	12	gaussian	constrained	-9.93	8.52E-15	1.53E-13	***
active_gm	12	gaussian	no smoothing	12.63	2.18E-19	3.92E-18	***
active_gm	12	constrained	no smoothing	10.98	1.25E-16	2.25E-15	***

5.2 Activation drift

Table S4. Parameter estimates and p-values for the GLMM describing the relationship between smoothing and the number of active voxels across regions as shown in Figure 7.

Region	Method	Parameter	Estimate	95% CI Lower	95% CI Upper	p value
GM	Constrained	Intercept	7.563	7.269	7.857	< 0.001
GM	Gaussian	Intercept	7.515	7.226	7.803	< 0.001
GM	Difference	Intercept	0.048	-0.055	0.151	0.359
GM	Constrained	Slope	0.014	0.004	0.024	0.006
GM	Gaussian	Slope	0.029	0.021	0.037	< 0.001
GM	Difference	Slope	-0.015	-0.028	-0.002	0.027
WM	Constrained	Intercept	6.966	6.536	7.396	< 0.001
WM	Gaussian	Intercept	7.009	6.611	7.407	< 0.001
WM	Difference	Intercept	-0.043	-0.358	0.272	0.788
WM	Constrained	Slope	0.043	0.011	0.074	0.008
WM	Gaussian	Slope	0.081	0.056	0.106	< 0.001
WM	Difference	Slope	-0.038	-0.078	0.002	0.063
RH Precentral	Constrained	Intercept	5.426	5.019	5.833	< 0.001
RH Precentral	Gaussian	Intercept	5.385	4.989	5.781	< 0.001
RH Precentral	Difference	Intercept	0.041	-0.14	0.221	0.659
RH Precentral	Constrained	Slope	0.022	0.004	0.04	0.018
RH Precentral	Gaussian	Slope	0.039	0.024	0.053	< 0.001
RH Precentral	Difference	Slope	-0.017	-0.04	0.006	0.149
RH Postcentral	Constrained	Intercept	5.441	5.15	5.732	< 0.001
RH Postcentral	Gaussian	Intercept	5.421	5.137	5.705	< 0.001
RH Postcentral	Difference	Intercept	0.02	-0.097	0.136	0.741
RH Postcentral	Constrained	Slope	0.024	0.013	0.036	< 0.001
RH Postcentral	Gaussian	Slope	0.041	0.031	0.05	< 0.001
RH Postcentral	Difference	Slope	-0.016	-0.031	-0.002	0.03

Table S5. Statistical comparisons (paired t-tests) for the results shown in Figure 8 analyzing the percentage of active voxels in the gray matter and postcentral gyrus.

Measure	FWHM	Mean Constrained	Mean Gaussian	Difference	t stat	p value
% active voxels in GM	3	66.81	62.33	4.48	9.08	< 0.001
% active voxels in GM	6	64.63	55.85	8.78	4.92	< 0.001
% active voxels in GM	9	64.21	53.48	10.73	4.39	< 0.001
% active voxels in GM	12	64.34	52.39	11.95	4.11	< 0.001
% active voxels in postcentral vs precentral	3	51.47	51.86	-0.38	-1.36	0.191
% active voxels in postcentral vs precentral	6	51.76	51.77	-0.01	-0.02	0.986
% active voxels in postcentral vs precentral	9	52.23	52.27	-0.04	-0.06	0.952
% active voxels in postcentral vs precentral	12	52.47	52.18	0.29	0.37	0.715

5.3 Activation reliability

Table S6. Parameter estimates and p-values for the GLMM describing the relationship between smoothing and the reliability of the resulting task activation maps.

Method	Parameter	Estimate	95% CI Lower	95% CI Upper	p value
Constrained	Intercept	0.350	0.250	0.450	< 0.001
Gaussian	Intercept	0.359	0.259	0.459	< 0.001
Difference	Intercept	-0.009	-0.025	0.007	0.270
Constrained	Slope	0.007	0.005	0.008	< 0.001
Gaussian	Slope	0.008	0.007	0.010	< 0.001
Difference	Slope	-0.002	-0.004	0.000	0.077

Table S7. Statistical comparisons (paired t-tests) for the results shown in Figure 9 analyzing the reliability of the activation maps after smoothing under both constrained (without resampling) and Gaussian smoothing.

Metric	FWHM	group1	group2	t stat	p value
Dice	3	Gaussian	Constrained	0.450	0.658
Dice	6	Gaussian	Constrained	0.366	0.719
Dice	9	Gaussian	Constrained	0.317	0.755
Dice	12	Gaussian	Constrained	0.277	0.785

6 RELATIVE SMOOTHING KERNEL WIDTHS

Table S8. Kernel width to voxel size ratios for each dataset. Since the voxel sizes varied between datasets, we have reported each smoothing kernel width relative to the voxel size of each dataset to facilitate comparisons of smoothing kernel widths between experiments.

Dataset	Voxel Size (mm)			Kernel Width / Voxel Size: FWHM = 3 mm			FWHM = 6 mm			FWHM = 9 mm			FWHM = 12 mm		
	x	y	z	x	y	z	x	y	z	x	y	z	x	y	z
Simulation	0.8	0.8	0.8	3.75	3.75	3.75	7.50	7.50	7.50	11.25	11.25	11.25	15.00	15.00	15.00
Sensory	1.7	1.7	4.0	1.76	1.76	0.75	3.53	3.53	1.50	5.29	5.29	2.25	7.06	7.06	3.00
Midnight Scan Club	4.0	4.0	4.0	0.75	0.75	0.75	1.50	1.50	1.50	2.25	2.25	2.25	3.00	3.00	3.00
HCP Aging	2.0	2.0	2.0	1.50	1.50	1.50	3.00	3.00	3.00	4.50	4.50	4.50	6.00	6.00	6.00
